# Energetic and Structural Insights into Water Confined in Hydrophobic Nanopores

**DOI:** 10.1021/acs.jpcc.5c08036

**Published:** 2026-01-22

**Authors:** Yuriy G. Bushuev, Alexander R. Lowe, Andrey Ryzhikov, Tomasz Wasiak, Michael Burt, Mirosław Chorążewski, Yaroslav Grosu

**Affiliations:** † Institute of Chemistry, University of Silesia in Katowice, 40-006 Katowice, Poland; ‡ Institut de Science des Matériaux de Mulhouse (IS2M), UMR 7361 CNRS, Axe Matériaux à Porosité Contrôlée (MPC), Université de Haute-Alsace, F-68100 Mulhouse, France; § Université de Strasbourg, F-67000 Strasbourg, France; ∥ The Chemistry Research Laboratory, Department of Chemistry, University of Oxford, Oxford OX1 3TA, U.K.; ⊥ Department of Chemistry, Trent University, Peterborough, Ontario K9L 0G2, Canada; # Centre for Cooperative Research on Alternative Energies (CIC energiGUNE), Basque Research and Technology Alliance (BRTA), Alava Technology Park, Albert Einstein 48, 01510 Vitoria-Gasteiz, Spain

## Abstract

Water confined within hydrophobic nanopores exhibits unusual thermodynamic and structural behavior that governs a wide range of nanofluidic and energy-conversion phenomena. Here, we combine high-pressure scanning calorimetry with molecular dynamics simulations in the temperature range of 298–380 K to elucidate the energetics and mechanisms of water intrusion into pure-silica LTA zeolites featuring cage-like pores. A new data-processing approach separates compression and intrusion contributions in pressure–volume curves, enabling direct quantification of temperature-dependent heat effects. Intrusion is exothermic at ambient temperature (≈−5 J g^–1^) and becomes nearly thermoneutral above 338 K. Simulations reveal slow intrusion kinetics, collective cage filling through hydrogen-bond-mediated chains, and pronounced stabilization at occupancies of 17–22 H_2_O molecules per supercell, balancing enthalpic and entropic factors. The results demonstrate that intrusion pressures correlate with accessible surface area, free pore volume, pore geometry, and connectivity rather than aperture size, thereby invalidating classical Laplace–Washburn scaling for nanopores. These findings establish microscopic design principles for tailoring wetting thermodynamics in hydrophobic nanoporous frameworks for energy storage, mechanical actuation, nanofluidic systems, and nanodevices.

## Graphical abstract

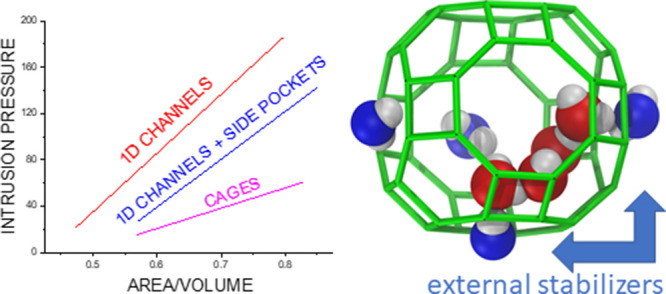

## 1 Introduction

Systems composed of water and hydrophobic porous solids represent a subclass of heterogeneous lyophobic systems (HLSs) exhibiting various physicochemical behavior.^1–3^ Water penetrates nanosized pores only under elevated pressure, referred to as the intrusion pressure (*P*_int_). Depending on the pore geometry and topology of the pore system, drying occurs at the extrusion pressure (*P*_ext_), which is equal to or lower than *P*_int_. These HLSs are, thus, categorized as either “molecular springs” or “shock absorbers”. Under certain conditions, a nonwetting liquid may remain in the pores after pressure release; such systems are termed “bumpers.”^4^

Hydrophobic solid matrices forming HLSs include pure silica zeolites (PSZs), metal–organic and covalent organic frameworks (MOFs and COFs),^5,6^ periodic mesoporous organosilicas,^7^ and grafted mesoporous silica materials, all of which can exhibit the three types of behavior described above. These systems have been explored for numerous applications,^8^ including energy storage and conversion,^4,9–11^ water purification and desalination,^12^ gas and liquid separation,^13^ and high-performance liquid chromatography.^14^ Key design parameters for tailored HLSs include the chemical stability of the solid matrix, the magnitude of intrusion/extrusion hysteresis, and the operational pressure range.

Such systems are also promising for nanotechnology and nanodevice fabrication, including micro/nanomotors^15,16^ and drug delivery vehicles.^17–19^ The hydrophobicity of mesoporous silica nanoparticles can be tuned through silane chemistry, and they are widely employed in biomedical applications due to their high adsorption and encapsulation capacities, biocompatibility, and biodegradability.^18^ At ambient pressure, when water cannot penetrate hydrophobic porous materials, colloidal aqueous suspensions of nanocrystals exhibit substantial gas adsorption capacity, opening new possibilities for practical applications.^20^ Consequently, a comprehensive understanding of the thermodynamic properties, intermolecular interactions, and structural modifications of confined water is essential.

Most experimental and theoretical studies of HLSs have been performed at ambient temperature.^3,21,22^ For practical applications, however, knowledge of the temperature dependence of their thermodynamic properties is crucial. The performance and efficiency of thermal machines based on wetting energy are governed by the heat effects associated with intrusion and extrusion and their temperature dependence.^23^ Such devices convert thermal energy into mechanical work.

A novel propulsion mechanism for micro- and nanoparticles — potentially applicable to nanorocket design — was proposed previously.^24,25^ It relies on the rapid expulsion of water from hydrophobic nanopores induced by microwave irradiation. The metastability of water near the extrusion pressure, altered structure under nanoconfinement relative to bulk water, and the spontaneous collective reorientation of molecular dipoles render confined water highly sensitive to weak external stimuli, triggering jet-like ejection. The work performed and heat released during extrusion propel porous micro- or nanoparticles.

Microwaves, as an energy source for nanomotors, offer significant advantages over light or ultrasound: they penetrate nonmetallic media, are highly biocompatible, and are inherently safe for biomedical applications.^26^ The development of nanodevices exploiting this propulsion mechanism, however, requires a comprehensive understanding of water behavior under nanoconfinement.

The wetting and drying of hydrophobic porous materials are complex phenomena influenced by multiple factors. Hydrophobic materials with channel-like and cage-like pores (channels and cages) demonstrate distinct behavior. In contrast to materials with channels, for cage structures, the intrusion/extrusion pressures depend strongly on the rate of pressurization.^27^ Experimental studies have shown that materials with channels absorb heat and mechanical energy during intrusion and release them upon extrusion.^28–30^ In contrast, materials with cages often display the opposite behavior.^25,31,32^ Simulations suggest that these differences originate from the structure of confined water.^25^ In globular clusters formed within cages, intermolecular interactions are stronger and molecular mobility is reduced compared to bulk water.

For hydrophobic nanotubes or zeolites with one-dimensional (1D) channels, it has been demonstrated that intrusion–extrusion hysteresis increases with pore width.^24^ The topology of zeolite frameworks, together with the geometry of the pores, strongly affects the pressures.^33^ Pressures can be tuned through chemical modification of internal channel surfaces, by altering pore connectivity,^33,34^ or by introducing hierarchical porosity^35,36^ — each approach modifying the operational pressure range.

PSZs with LTA, CHA, and SOD topologies consist of cage-type frameworks. Here and throughout this work, we use the three-letter codes to denote framework topologies and cage types, following the nomenclature adopted by the Structure Commission of the International Zeolite Association (IZA-SC).^37^ Framework fragments of these zeolites are presented in Figure 1. LTA zeolites with various chemical compositions are widely employed in commercially relevant technologies.^38^ The present study focuses on ITQ-29, a synthetic pure silica material with LTA topology and a unit cell composition of (SiO_2_)_2__4_.^39^ The LTA structure shown in Figure 1 consists of sodalite (β) cages with an average diameter of 6.6 Å, which are connected through double four-member rings (*d4r*) to form supercages (α-cages) with a diameter of 11.4 Å.^40^ Each supercage is surrounded by eight sodalite cages (*sod*), forming a cubic framework. Six-membered ring (6 MR) windows connect the supercages with the sodalite cages, and the supercells are interconnected in three perpendicular directions through 8 MR windows.

**Figure 1:**
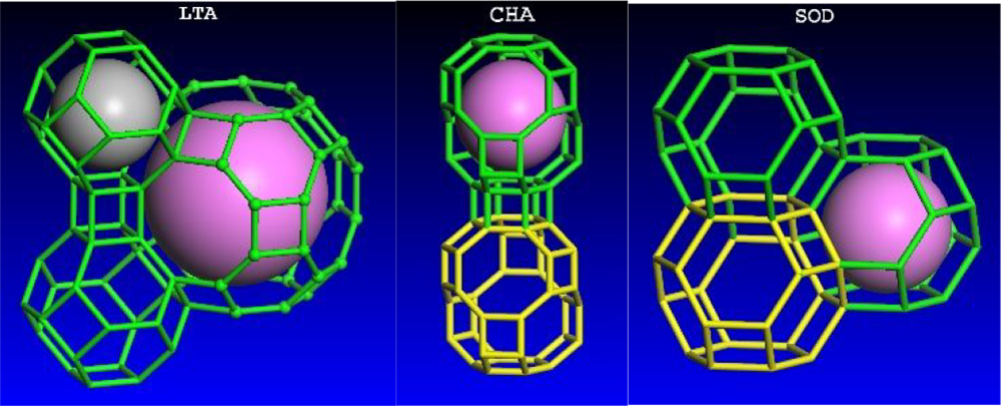
Structural fragments of the LTA, CHA, and SOD frameworks. For LTA, one supercell, two sodalite cages, and a connecting double four-membered ring (*d4r*) are shown. For CHA, two identical *cha* cages connected by a double six-membered ring (*d6r*) are depicted. For SOD, three identical *sod* cages are displayed. Spheres located within the cages highlight cage geometry. Silicon atoms occupy the vertices of the polyhedra and are connected by lines; oxygen atoms are omitted for clarity.

Numerous studies have focused on the hydrophilic LTA-type zeolites, such as 4A, which has the unit cell formula [Na_12_(H_2_O)_2_7]_8_[Al_12_Si_12_O_4__8_]_8_, 5A, and related materials.^41–45^ In contrast to water adsorption in hydrophilic zeolites,^46^ investigations of the water intrusion and extrusion are more complex. These processes occur under high pressure, requiring specialized equipment. The synthesis of hydrophobic materials presents additional challenges. Consequently, water intrusion/extrusion isotherms have been obtained for only a limited set of PSZs.^3,4,22^ Water intrusion in ITQ-29 has been studied experimentally^47,48^ and via computer simulations,^25,49–51^ predominantly at ambient temperature.

ZIF-8, a metal–organic framework, exemplifies an important class of porous materials characterized by cage-like pores.^11^ Its structure adopts the SOD topology,^18^ as illustrated in Figure 1. In contrast to SOD-type PSZ, the presence of organic linkers increases the distance between metal atoms at the vertices of the polyhedral cages. As a result, the pore apertures are wider than in sodalite cages of zeolites. The framework of ZIF-8 is more flexible than that of typical zeolites. In this case, water penetrates through 6MR windows that interconnect the cages. During water intrusion, ITQ-29 (LTA),^25^ chabazite (CHA),^30^ and ZIF-8 (SOD)^31^ all exhibit exothermic behavior at ambient temperature. ITQ-29 demonstrates “bumper” behavior, in which water remains confined within the pores after pressure release, whereas the water–ZIF-8 and water–CHA systems act as shock absorbers, showing prominent hysteresis in the intrusion/extrusion *P*–*V* diagrams. The temperature dependence of the heat effects has been established for ZIF-8: the exothermic heat decreases with increasing temperature, and the process becomes endothermic above 340 K.^31^ For other microporous materials, however, the temperature dependence of heat effects remains unexplored.

Computer simulation methods provide detailed insights into atomic-level dynamics, interparticle interactions, thermodynamic properties, and intrusion/extrusion isotherms of HLSs.^4,31,51–53^ However, these simulations are limited by finite system size, short time scales, and uncertainties in interaction energies. Nevertheless, they serve an essential role in rationalizing and systematizing experimental data, identifying correlations, and predicting the properties of HLSs.^33^

This study reports systematic experimental investigations of HLSs composed of water and LTA-type PZS at 298.15, 318.15, and 338.15 K. Two different samples of zeolite were examined. Intrusion isotherms and intrusion heats were measured using a scanning transitiometer at the University of Silesia in Katowice (Poland). Complementary computer simulations were conducted at 300, 340, and 380 K to rationalize the experimental findings and further elucidate the thermodynamics of water intrusion across a broad range of pressures and temperatures. Comparison with nanoporous materials featuring cage- and channel-like pores enabled a generalization of the findings and revealed the key differences between these systems.

## 2 Materials and Methods

### 2.1 Scanning Transitiometry (High-Pressure Scanning Calorimetry)

Two pure-silica LTA-type zeolite (ITQ-29) samples were synthesized via different routes. The first (ITQ) was prepared and characterized at ITQ, Valencia, Spain,^39,54^ and the second (MUL) at the Mulhouse Materials Science Institute (IS2M), France.^47,48,55^ Characterization details are available in the cited works, with additional analyses provided in the Supporting Information (Table S1 and Figures S1–S3).

High-pressure calorimetric measurements were performed using a BRG-tech scanning transitiometer (University of Silesia, Katowice), described previously.^56^ The instrument allows simultaneous recording of pressure–volume isotherms and differential heat flow. A weighed mass of LTA zeolite was loaded into stainless steel cylindrical tubes (outer diameter 6 mm, wall thickness 0.5 mm) sealed with fiberglass plugs. Prior to measurements, the samples were regenerated by heating at 550 °C for 13 h, followed by evacuation (4 × 10^–4^ mbar overnight).

The sample was placed in a calorimetric measuring cell connected to a manifold and a high-pressure line. Both the measuring and reference cells were filled with deionized water, mounted in parallel on the same line, and sealed under controlled torque conditions.

Calorimetric experiments were conducted at 298.15, 318.15, and 338.15 K. The transitiometer was equilibrated for 90 min at the target temperature before pressurization. Pressure was increased at a constant rate of 0.5 MPa min^–1^ up to 40 MPa using a stepper-motor-driven piston. Differential heat flux between the measuring and reference cells—both filled with water—was continuously recorded and converted to specific thermal power, along with simultaneous monitoring of pressure and volume changes. The total duration of each experiment was approximately 3.5 h.

### 2.2 Molecular Dynamics Simulations

Molecular dynamics (MD) simulations of water–PSZ systems were performed using fully flexible zeolite frameworks and flexible water molecules. Numerous force fields (FFs) have been developed for atomistic simulations of zeolites,^57,58^ reflecting the challenge that no single FF can accurately reproduce all zeolite properties. Moreover, the transferability of a given FF between different topologies is generally limited.^59,60^ In this work, the BS force field,^52,61,62^ consisting of electrostatic, van der Waals, and three-body interaction terms, was employed for zeolite energy calculations. This FF has been shown to reproduce experimental enthalpies of PSZs at 300 K with high accuracy and to provide reliable structural parameters of the frameworks.^63^

Water molecules were described by the SPC/Fw model, which allows for intramolecular flexibility.^64^ Electrostatic interactions were treated with the Ewald summation method, while short-range interactions were computed directly with a cutoff distance of 9 Å, as recommended in the original study.^64^ All van der Waals interactions were evaluated in real space with the same cutoff (∼2.5σ). Long-range corrections were applied. Parameters and functional forms of the force fields, together with input files for GULP and DL_POLY, are provided in the Supporting Information.

Two complementary MD procedures were employed:

Procedure 1. The General Utility Lattice Program (GULP) v6.2.0 was used to simulate water–zeolite systems over a wide range of water loadings.^65,66^ Each computational cell consisted of a single crystallographic unit cell of the zeolite and *N*_*w*_ water molecules, with periodic boundary conditions. Simulations were performed in the NPT ensemble at *T* = 300, 340, and 380 K and *P* = 100 MPa for isotropic cells. The integration time step was 1 fs; equilibration and production runs lasted 1 and 2 ns, respectively.

Procedure 2. The DL_POLY v5.1.0 code^67^ was used to model ITQ-29 (LTA) zeolite immersed in bulk water. The cubic simulation box contained a zeolite grain with 64 α-cages (supercells) and 30,000 water molecules. Periodic boundary conditions were applied to eliminate surface effects. Simulations were conducted in the NPT ensemble at *T* = 300 and 380 K and *P* = 0–150 MPa using the Melchionna modification of the Nosé–Hoover algorithm, with relaxation times of 1 ps (thermostat) and 5 ps (barostat). The integration time step was 1 fs, and production runs extended up to 20 ns. Electrostatic interactions were calculated using the Smooth Particle Mesh Ewald (SPME) method, with the short-range part computed in real space up to 9 Å and the long-range part in reciprocal space.

## 3 Results

Experimental measurements and molecular dynamics (MD) simulations of the LTA-type PSZ–water system were performed to elucidate the mechanism of water intrusion in cage-type nanoporous materials, as well as the associated energetics and structural transformations of confined water. Each method has inherent limitations and uncertainties; however, when combined, the experiments and simulations provide a consistent atomistic interpretation that enables predictions for related systems.

### 3.1 Experiments

LTA-type PSZs synthesized, at the Instituto de Tecnologia Quimica (ITQ) and at the Institut de Science des Matériaux de Mulhouse (IS2M), following different protocols, are hereafter referred to as ITQ and MUL.^39,47,55^ Numerical indices following these abbreviations correspond to the number of completed intrusion cycles for a given sample. Pressure, volume, and thermal power were monitored over time using a scanning transitiometer (PVT calorimeter).^68,69^

Intrusion/extrusion isotherms are characteristic features of HLSs. For both the ITQ and MUL samples of the ITQ-29 material, only intrusion was observed, indicating bumper-type behavior of the LTA–water systems. After intrusion, high-temperature treatment was required to restore the material and enable repetition of the experiment.

Unlike standard porosimetry, the intrusion process recorded with a transitiometer is strictly isothermal and substantially slower, which may account for discrepancies between intrusion P–V isotherms obtained by different experimental techniques. Additionally, the pressurization rate is computer-controlled and kept constant, with the stepper motor adjusting the rate of volume change accordingly. The volume changes recorded during the transitiometer intrusion experiments arise from several contributions: (i) the intrusion of water into the porous framework, (ii) the compressibility of bulk water in the twin cells, (iii) the compressive response of the zeolite matrix, both empty and filled, and (iv) the compressibility of oil and mercury within the pressure transmission line connecting the piston with the measuring and reference cells.

Water intrusion is a rapid event that occurs within a narrow pressure interval, whereas the compression-related contributions evolve more gradually. Consequently, the recorded volume in time response represents a superposition of a fast (intrusion) and a slow (compression) process. To decouple these contributions, the experimental volume response, *V*_exp_, was fitted with a combination of a polynomial and a sigmoidal dose–response function:

$$V_{\exp}\left(t\right)=a+bt+ct^{2}+\frac{p}{\left[1+10^{\left({\log x}_{0}-t\right)h}\right]}$$ 1

where *a, b,* and *c* are adjustable parameters of the polynomial term, and *p*, log*x*_0_, and *h* are adjustable parameters of the dose–response function. The results of the fitting procedure, along with statistical indicators of fit quality, are presented in Figures 2a.

**Figure 2:**
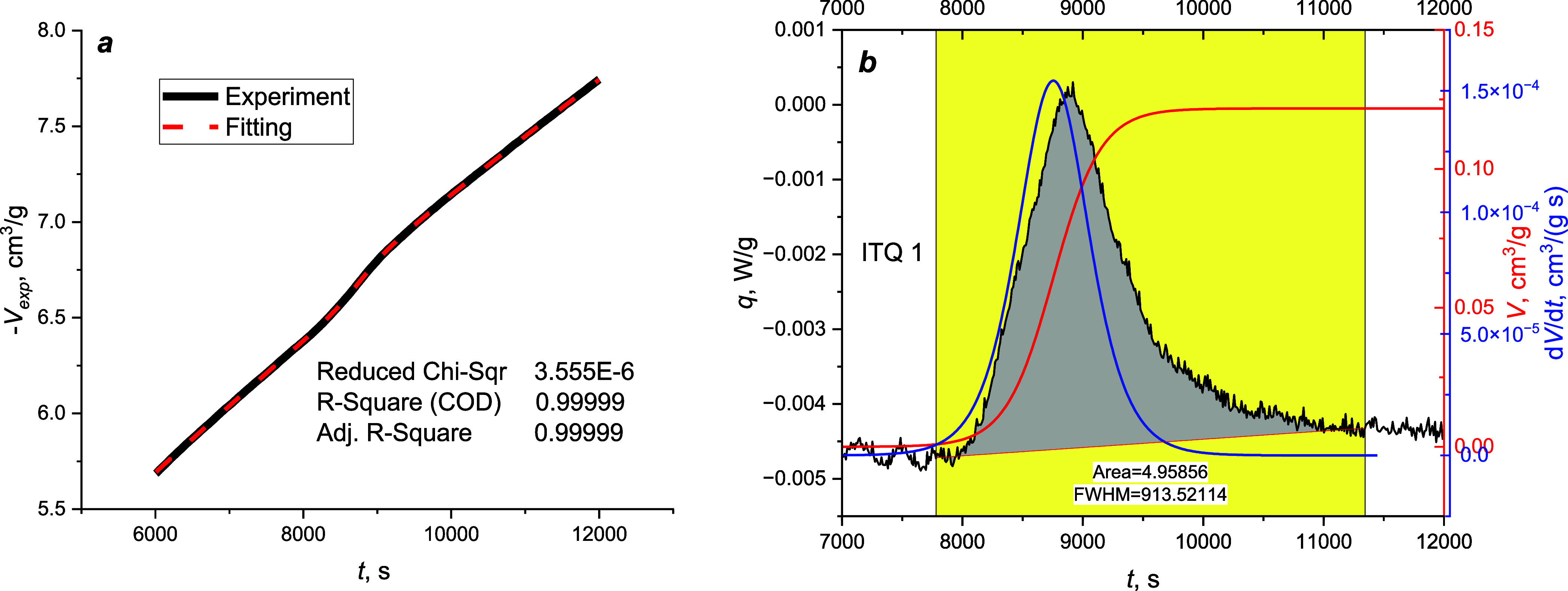
Time evolution of (a) the apparent volume (*V*_exp_) of intruded water in ITQ-29 at 298 K, together with the fitting results obtained using eq 1; (b) specific thermal power *q* (black line), intruded volume *V* (red line), and the first derivative d*V*/d*t* (blue line).

Figures 2b and S4 show the typical time evolution of the specific thermal power (*q*) recorded by the transitiometer, together with the volume of intruded liquid (*V*) and the intrusion rate (d*V*/d*t*). The function *V*(*t*) was calculated using only the dose–response component of eq 1. The peak in the intrusion rate precedes the peak in heat production *q*, consistent with the Tian equation.^70^ The time constant in the Tian equation characterizes the thermal inertia of the system. The specific heat of intrusion (*Q*) was determined as the integral of the power curve, *Q* = ∫*q*(*t*)­d*t*, corresponding to the area enclosed between the signal curve and the line connecting the initial and final baselines.

Figure 3a–c present intrusion isotherms at three temperatures, plotted as *P*–*V* curves. For this purpose, the calculated *V*(*t*) from eq 1, together with the recorded pressure function *P*(*t*), were used. By definition, the intrusion pressure *P*_intr_ corresponds to the pressure at which half of the maximum volume is intruded. The original data were normalized as *V*(*t*)/*V*_max_, where *V*_max_ = *p* in eq 1. Additionally, Figure 3d–f show the same data expressed as fractional loadings.

**Figure 3:**
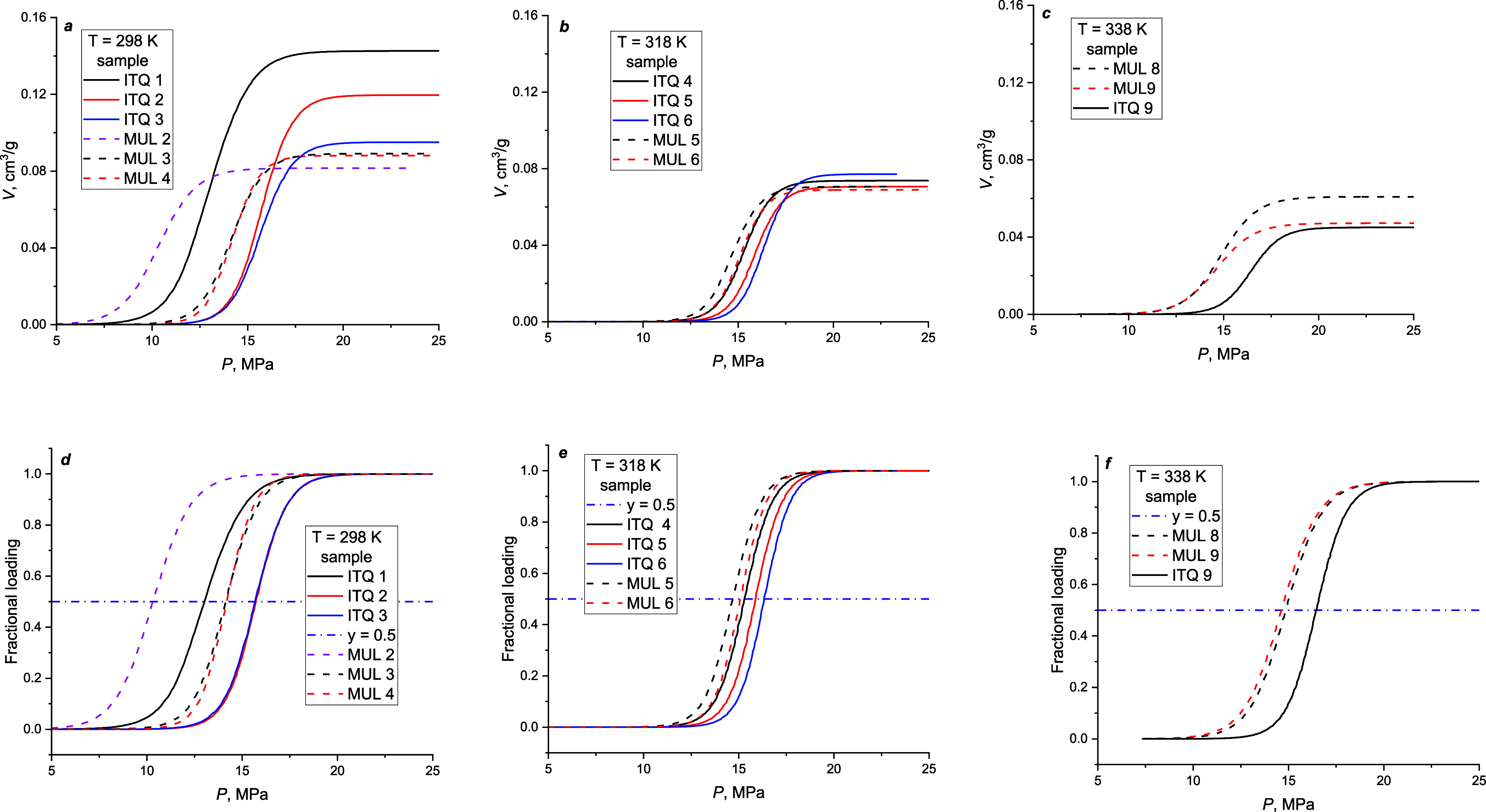
Intrusion isotherms of LTA-type pure-silica zeolites at different temperatures, shown as pressure–volume curves (a–c) and fractional loading–pressure plots (d–f). ITQ and MUL indicate the origins of the sample, and the digit denotes the number of the intrusion–recovery cycle.

The as-synthesized ITQ 1 sample exhibits slightly different properties compared to samples subjected to multiple intrusion and thermal treatment cycles, specifically, lower *P*_intr_, and higher *V*_max_. The chemical stability of the material plays a significant role: progressive degradation predominantly affects fresh materials and slows down with successive intrusion–recovery cycles.^3,47^ A decrease in water intrusion volume accompanies material degradation. It is difficult to disentangle the effects of degradation from the thermodynamic reduction of water content in the pores. Both factors act in the same direction, causing a significant decrease in *V*_max_ with increasing temperature. Figure 3 demonstrates that the slopes of the curves, expressed as fractional loading versus pressure, are nearly the same, suggesting that the mechanism of water intrusion is insensitive to temperature.

Examples of differential scanning curves *q*(*t*) presented in Figures 2b and S4 illustrate the differences in heat effects recorded at two temperatures. The heat of intrusion decreases with increasing temperature, which complicates accurate determination due to large signal fluctuations near the detection limit of the device. In some cases, it was not possible to reliably calculate the heat.

Figure 4 shows a progressive decrease in heat with increasing temperature. Because the statistical uncertainties are relatively large, multiple experiments are necessary to obtain more reliable average values, especially when the heat effects are small. Sample degradation also influences the measured heat: hydrolysis of crystals, resulting in the formation of silanol defects, is accompanied by additional heat effects that are difficult to separate and model. Figure 4b shows heat effects normalized per volume of intruded water. In this case, differences in intrusion volume between experiments, as shown in Figure 3, are accounted for, reducing the influence of zeolite degradation.

**Figure 4:**
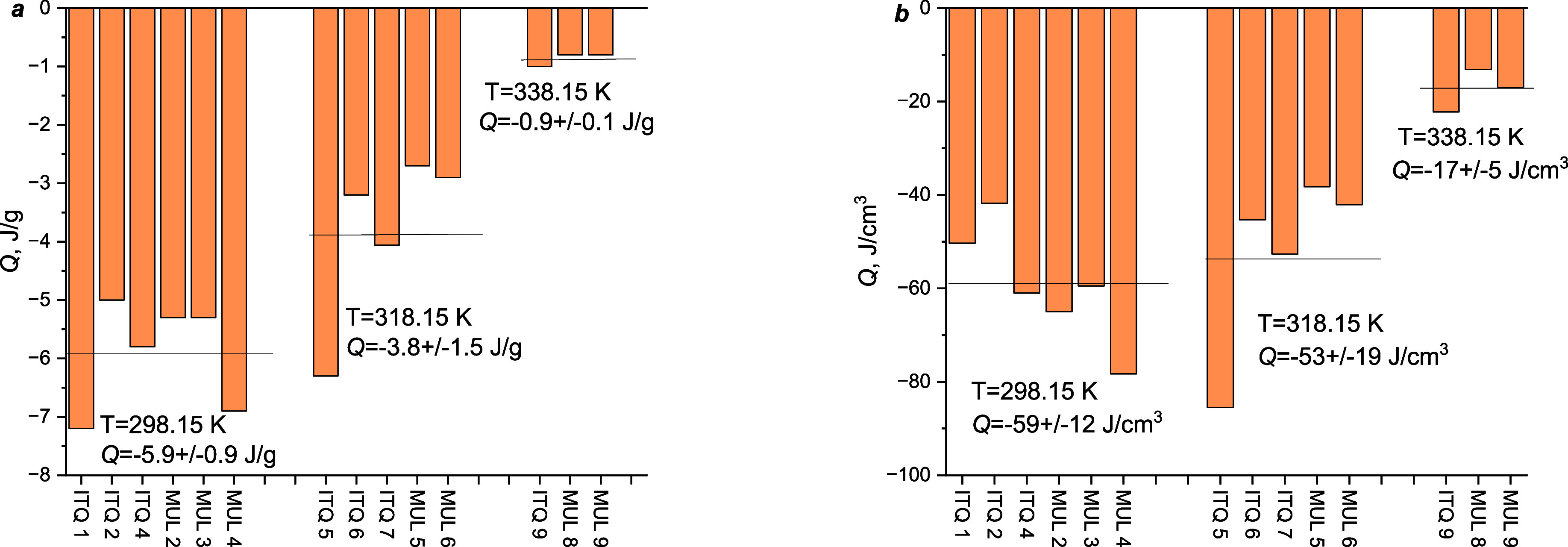
Experimental heats of intrusion for two LTA-type pure-silica zeolite samples in the range 298–338 K: (a) normalized per gram of zeolite; (b) normalized per cm^3^ of intruded water.

### 3.2 Computer Simulations

#### 3.2.1 Thermodynamics

The GULP program^66^ was employed to calculate the energetics of the LTA–water system at various temperatures according to Procedure 1 (see Methods). The specific energies for transferring *N*_*w*_ water molecules from bulk water into the zeolite, Δ*U*, were calculated according to eq 2:

$$\Delta U=U_{wz}-U_{w}-U_{z}$$ 2

where *U*_*wz*_ is the internal energy of the LTA–water system, *U*_*w*_ is the energy of *N*_*w*_ water molecules calculated from independent simulations of bulk water, and *U*_*z*_ is the energy of the empty zeolite.

We adopted the BS force field for the zeolite^61–63^ and SPC/Fw for water.^64^ These force fields have been widely employed in simulations of water–zeolite systems, and the calculated intrusion/extrusion pressures and intruded water volumes are in good agreement with experimental values.^24,34,52,53^ Comparisons of these results with those obtained using other force fields, and their dependence on variations in BS force field parameters, have been discussed previously (see SIs).^34,52^

The isosteric heat of adsorption, *Q*_st_, is defined as the difference in the partial molar enthalpy of the sorbate between the gas phase and the adsorbed phase.^41,53,71^ If the energy is calculated per molecule, then:

$$Q_{\mathrm{st}}=k_{B}T-\left(\frac{\partial U_{wz}}{\partial N_{w}}\right)$$ 3

The *U*_*wz*_(*N*_*w*_) values presented in Figure 5a were fitted with polynomials, and derivatives were subsequently calculated. The resulting *Q*_st_ curves, presented in kJ/mol, are shown in Figure 5b together with experimental data for hydrophilic zeolite 4A (NaA).^41^ Additional experimental and calculated data for Na-LTA zeolites are available in the literature.^43^ Despite differences in hydrophobicity, the heats of adsorption are approximately the same at *N*_*w*_ ≈ 20. In hydrophilic zeolites, water interacts strongly with atoms in the framework and extra-framework cations, leading to higher heats of adsorption. However, at high loadings, newly added molecules interact primarily with other water molecules rather than with ions. The agreement with experimental values indicates that the computational model is adequate.

**Figure 5:**
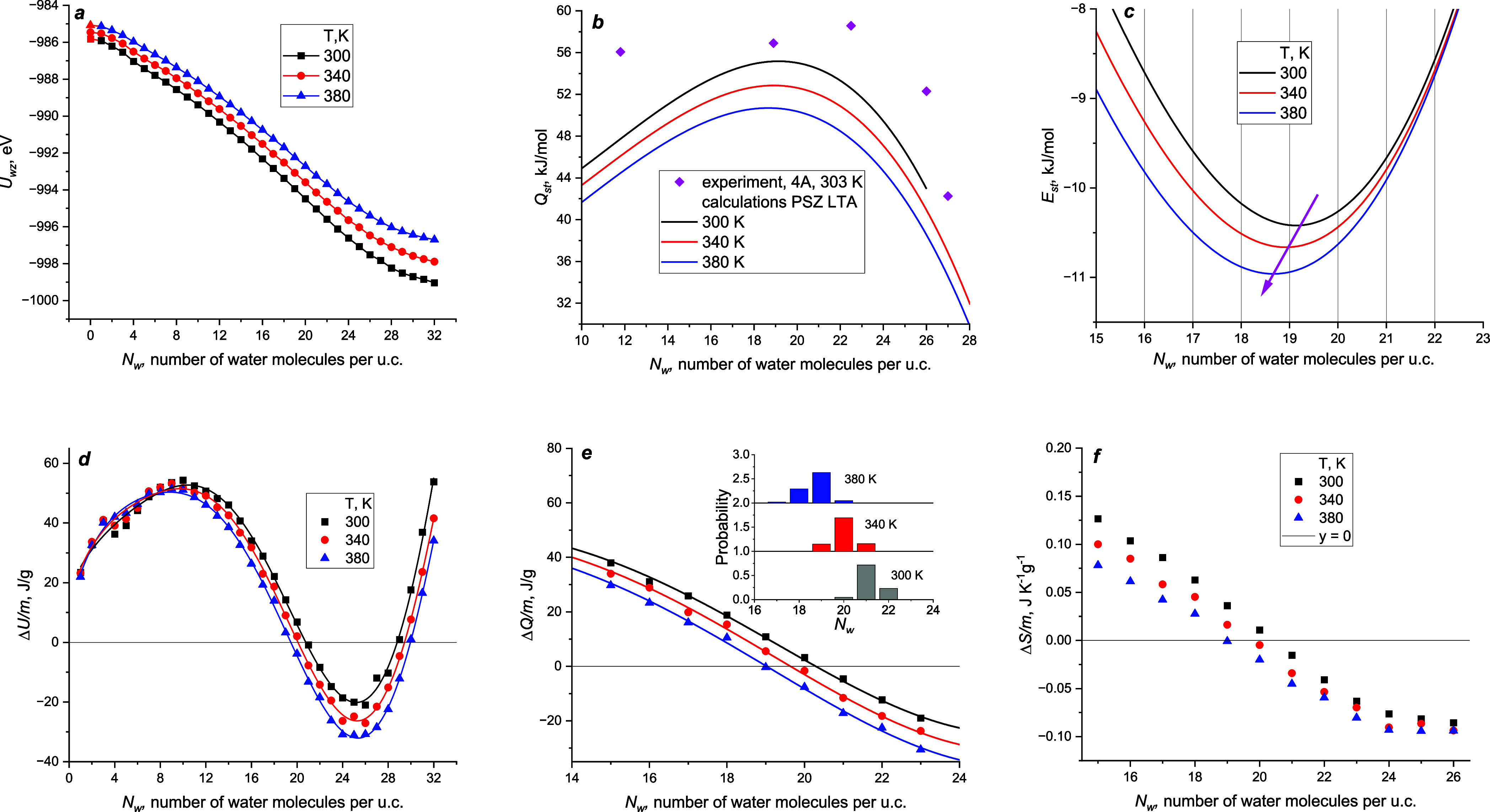
Thermodynamics of water intrusion into LTA: (a) potential energy of the water–zeolite system; (b) experimental isosteric heat of adsorption for hydrophilic 4A zeolite (LTA) and calculated values for hydrophobic pure silica LTA; (c) isosteric energy of intrusion; (d) changes in specific excess internal energies; (e) heats of intrusion and probabilities of water occupancies (inset); (f) entropies for water transfer from bulk water into the zeolite.

Adsorption and intrusion are distinct processes. During intrusion, the water confined within the pores is in equilibrium with the bulk liquid phase rather than with vapor. By analogy with eq 3, we propose calculating the isosteric energy of intrusion as

$$E_{\mathrm{st}}=\left(\frac{\partial U_{wz}}{\partial N_{w}}\right)-U_{w}$$ 4

Negative values of *E*_st_ indicate the stabilization of water within the pores relative to bulk water, corresponding to the transfer of a single molecule from the liquid phase into a pore containing *N*_*w*_-1 molecules. The calculated stabilization energies are shown in Figure 5c. The magnitude of stabilization is approximately 10 kJ mol^–1^, and the absolute values of *E*_st_ increase with temperature. The minimum of the *E*_st_(*N*_*w*_) function occurs at *N*_*w*_ ≈ 20 and shifts toward lower loadings as the temperature increases.

The mechanical work, *W*, required for water intrusion into the zeolite is given by *P*_int_ × Δ*V*, where Δ*V* = *V*_max_ represents the change in the volume of intruded water. The corresponding energy balance for an intrusion is expressed by eq 5 ^1,25,30^ :

$$\Delta Q=\Delta U-P_{\mathrm{int}}\Delta V_{.}$$ 5

Results of the Δ*U* and Δ*Q* calculations are presented in Figure 5d,e and S5a, covering the full range of water loadings. These quantities represent excess thermodynamic functions that characterize the overall effect when *N*_*w*_ water molecules are transferred from the bulk liquid phase into the pores. The strongest energetic stabilization is observed when approximately 22–26 water molecules occupy the LTA supercell, and this effect becomes more pronounced at elevated temperatures. The free energy governs the intrusion process. A decrease in internal energy may be accompanied by a reduction in system entropy, which compensates for the energetic term. When water in both phases is at equilibrium, Δ*G* = 0 and Δ*Q* = *T*Δ*S*. However, based on the curves shown in Figure 5e,f, it is not possible to determine the exact *N*_*w*_ at which intrusion occurs.

Figures 5f and S5b (for all loadings) show that the entropy change becomes negative for *N*_*w*_ > 20, indicating that water inside the pores is more ordered than in the bulk phase. A decrease in the entropy of water in solvation shells is a typical signature of hydrophobic solutes.^72^ When water forms globules within the hydrophobic cage-like nanopores of LTA, both energetic and entropic stabilization are observed.

Intrusion occurs spontaneously if Δ*G* < 0, that is, when *P*Δ*V* > Δ*U*-*T*Δ*S,* or equivalently, when *P* > *P*_*int*_, where Δ*V* is the volume of the intruded water. This condition is satisfied at a loading of approximately 20 molecules per unit cell. The behavior of the presented curves does not allow direct estimation of the amount of intruded water in actual experiments, since fluctuations in *N*_*w*_ are not permitted within the adopted computational scheme. To resolve this limitation, free-energy calculations or direct molecular simulations of intrusion would be required.

Grand Canonical Monte Carlo (GCMC) calculations of the Landau free energy revealed that the second minimum on the curve, corresponding to the stable state of filled pores, appears at approximately 70 MPa and is located near *N*_*w*_ = 20.^51^ At lower loadings or pressures, water confined in the pores resides in a metastable state. The reported pressure substantially exceeds the experimental intrusion pressure; however, this discrepancy depends on the quality of the model and the employed force fields.

According to the GCMC results, the unit cell of a pure-silica LTA-type zeolite, containing one supercage and one *sod* cage, can accommodate about 21 water molecules.^51^ It has been shown that up to four water molecules can penetrate *sod* cages when the zeolite is hydrophilic, as in the case of NaY with the FAU topology, which also contains *sod* cages.^73^ However, with increasing hydrophobicity of the LTA framework (i.e., at higher Si/Al ratios),^74^ water tends to migrate out of the *sod* cages.^75^

We employed an alternative approach (Procedure 2), which involved direct molecular simulations of water intrusion into a cubic crystalline grain composed of 64 LTA supercells. The grain was immersed in liquid water. The simulations demonstrate that, even at high pressures, water molecules cannot pass through the narrow 6MR windows of the *sod* cages. No water intrusion was observed experimentally in pure-silica SOD-type zeolite, which contains only *sod* cages. Nevertheless, GCMC calculations predict the presence of some water molecules inside these cages, since the method relies on random insertion and deletion of molecules in both cage types. Consequently, in hydrophobic LTA zeolite, water molecules are unable to penetrate through the 6MR windows, which effectively prevents their entry into the sodalite pores.

Our simulations revealed several characteristic features. Water readily fills the supercells in contact with the bulk phase even at ambient pressure; slow kinetics are observed for filling the eight central cages within the grain; and, over a range of pressures, some of the core cages remain unfilled. It has been shown that, depending on the position of a pore within the crystal, pores may fill at different pressures.^34,36^ Larger grains and longer simulation times are required to determine the intrusion pressure accurately. This situation is considerably more complex than in zeolites with channel-like topologies, since neighboring supercells in LTA are separated by 8MR windows, and water molecules must overcome significant energetic barriers.^51^

We further calculated the distributions of water molecules within the supercages at three temperatures under 100 MPa, when all supercells in the grain core were filled with water. The resulting distributions, presented in Figure 5e, indicate that each supercell accommodates approximately 17–22 water molecules. These distributions are narrow, and their maxima shift toward lower occupancies with increasing temperature. In this temperature range, variations in internal energy and entropy are minimal (Figure 5d,f).

We note that the *E*_*st*_ curves in Figure 5c exhibit similar behavior, with their minima occurring in the same range of loadings (16 < *N*_*w*_ < 22) and shifting toward smaller values with increasing temperature. Thus, Procedure 1 may be used to estimate loadings after intrusion without requiring complex calculations. The heat of intrusion was calculated according to the following equation:

$$Q=\Sigma P\left(N_{w}\right)\times \Delta Q\left(N_{w}\right)$$ 6

where *P*(*N*_*w*_) is the probability of various water occupancies in the supercell, and Δ*Q*(*N*_*w*_) is presented in Figure 5e.

#### 3.2.2 Hydrogen Bonding

Hydrogen bond statistics provide a quantitative measure of structural changes in water as a function of temperature and pore occupancy. However, any definition of hydrogen bonding in liquid water is to some extent arbitrary.^76^ In this work, two criteria were employed to identify hydrogen bonds. The energetic criterion defines two molecules as bonded if their interaction energy is below −3.5 kcal/mol (*E*_HB_ < −14.64 kJ/mol).^25,36^ The geometric criterion considers molecules bonded if the oxygen–oxygen distance (*R*_OO_) is less than 3.3 Å and simultaneously the oxygen–hydrogen distance (*R*_OH_) is below 2.41 Å.^25,76^ Each of these criteria emphasizes different aspects of the transition of water from the bulk phase into pores.

The results of the calculations are summarized in Figure 6. As expected, the number of H bonds, *N*_HB_, decreases with increasing temperature and increases with pore loading (Figure 6a). At *N*_HB_ > 25, the close packing of molecules renders the functions obtained with the geometric criterion nearly temperature-independent. In contrast, with the energetic criterion, the functions reach distinct plateaus.

**Figure 6:**
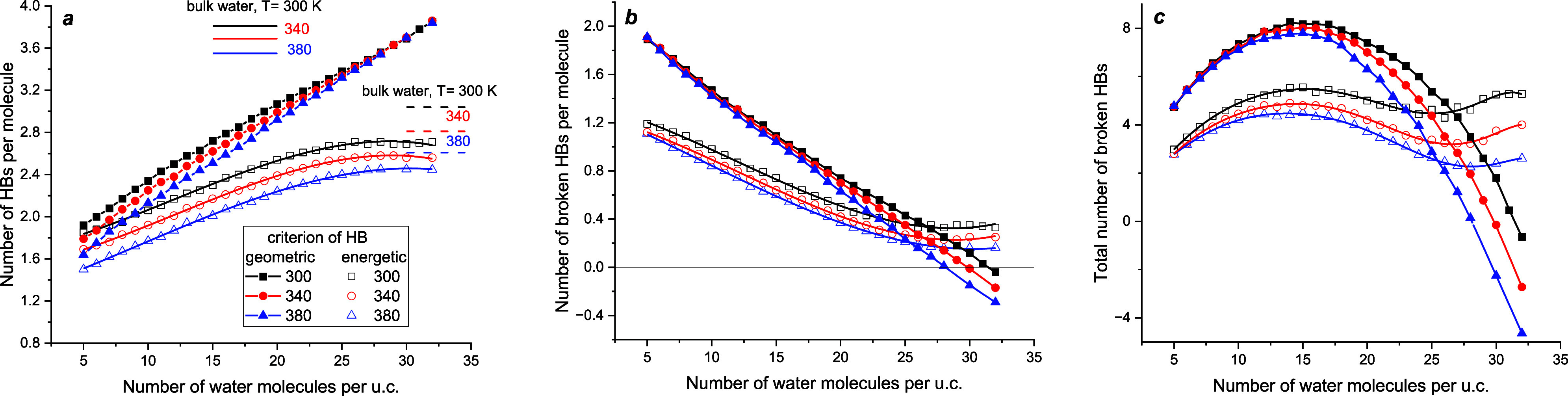
Statistics of hydrogen bonds in water intruded into LTA-type pure-silica zeolites, calculated using geometric and energetic criteria: (a) average number of H-bonds per water molecule, (b) number of broken H-bonds per water molecule, and (c) total number of broken H-bonds as a function of the number of water molecules per unit cell. Solid symbols represent results obtained using the geometric criterion, while open symbols correspond to the energetic criterion.

The number of broken bonds per molecule is defined as the difference between the average number of bonds in bulk water and *N*_HB_ in pores calculated per molecule. These functions are shown in Figure 6b. The number of broken bonds progressively decreases with increasing loading. At elevated temperatures, it is lower than in bulk water. Negative values, observed at high loadings using the geometric criterion, indicate the formation of additional bonds due to close packing.

The total number of broken bonds was calculated as *N*_all_ = *N*_*w*_ × *N*_HB_/2, since each H bond connects two molecules. The corresponding curves are shown in Figure 6c. For the energetic criterion, the curve shape resembles that in Figure 5d, but with a stronger temperature dependence. The functions reach maxima at *N*_*w*_ ≈15, indicating the barriers between the empty and filled pore states.

Hydrogen bond statistics, which characterize the structure of water, account for only nearest-neighbor interactions. According to both criteria, at *N*_*w*_ < 25, water loses hydrogen bonds upon transfer into pores due to the formation of an interface with the solid. Figure 6 shows that the structural properties of water in pores depend on their occupancy. Consequently, the effective surface tension — or, more precisely, the excess interfacial free energy — of water nanodroplets confined within cage-like pores depends on both the pore diameter and the degree of occupancy, as well as on temperature.

#### 3.2.3 Mobility of Water inside LTA PSZ

The mobility of confined water was evaluated through self-diffusion coefficients (*D*).^77^ For three-dimensional Fickian diffusion, Einstein’s relation applies,

$$R^{2}\left(t\right)=6Dt$$ 7

where *R* ^2^ is the mean square displacement (MSD). Computational details are given elsewhere.^25^ Figure 7 shows the self-diffusion coefficients at three temperatures as functions of pore loading. For clarity, the bulk water coefficient at 380 K (*D* = 7.4 × 10^–9^ m^2^ s^–1^) is not included. In all cases, confinement leads to a large reduction in mobility relative to bulk water, most strongly at 380 K. In contrast, temperature plays a secondary role for confined water. The highest diffusivity is observed in the range 8 < *N*_*w*_ < 17, which coincides with the maximum in the number of broken hydrogen bonds (Figure 6c) and the excess internal energy (Figure 5d). At higher occupancies *N*_*w*_ > 20, the diffusivity drops sharply. This reduction hinders penetration of water into the zeolite interior.

**Figure 7:**
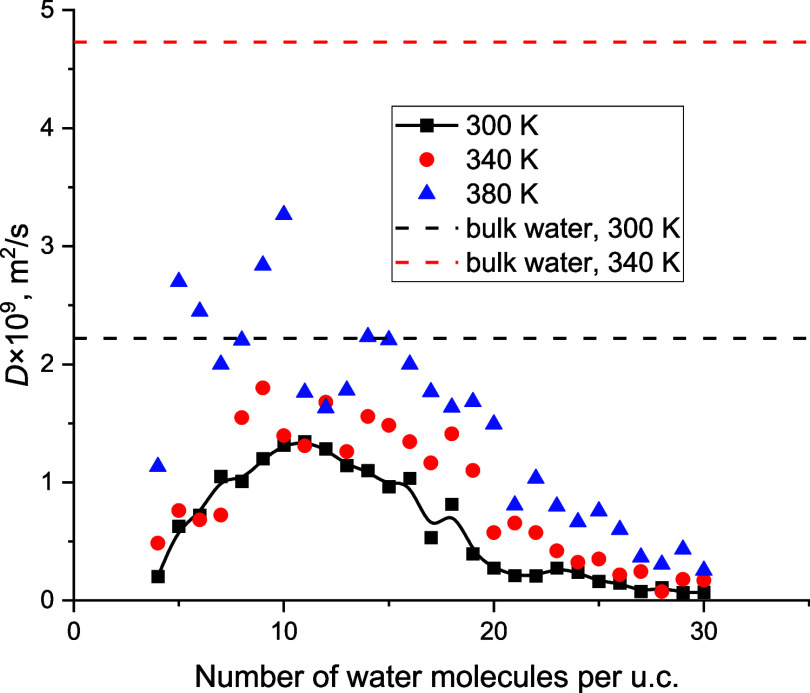
Self-diffusion coefficients of water in LTA-type PSZ at three temperatures. The curve is shown as a guide to the eye.

## 4 Discussion

### 4.1 Intrusion/Extrusion Isotherms

Most reported intrusion/extrusion isotherms have been obtained using porosimetry equipment, where intrusion occurs rapidly (5–10 MPa min^–1^).^3,4^ In contrast, the transitiometer records a slow, essentially isothermal quasi-static process (0.5 MPa min^–1^) with pauses at the final pressure to allow for thermal equilibria. For ZIFs with cage-type structures, it has been shown that high pressurization rates lead to nonequilibrium intrusion, shifting *P*_int_ to higher values.^27,78^ Slower pressurization allows the system to relax toward equilibrium, yielding lower measured *P*_int_. This kinetic dependence highlights the metastable nature of confined water and its sensitivity to experimental time scales. In contrast, PSZs with channel-type pores exhibit a much weaker rate dependence of *P*_int_.^27^ Accordingly, the 5–10 MPa discrepance between porosimetry and transitiometry data for LTA may partly originate from differences in the pressurization rates applied in these techniques.

Another origin of the discrepancy is the slightly different properties of the ITQ and MUL samples. Characterization of ITQ is presented in the Supporting Information. The diffractograms (Figure S1) reveal an increased fraction of amorphous phase and a 19% decrease in pore volume (Table S1 and Figure S2) after the transitiometer experiments. The amorphous phase fraction decreases upon subsequent heating at 330 °C. Thermogravimetric analysis (Figure S3) shows that the ITQ sample is less hydrophobic than MUL and that the number of silanol groups increases after water intrusion. Overall, the LTA-type zeolite exhibits the lowest *P*_int_ among the investigated PSZs,^22^ and the discrepancy is not significant for most practical purposes.

During each transitiometer experiment, thermal power and incremental volume changes in the twin cells were recorded simultaneously. Although the heat associated with bulk water compression is effectively subtracted, the observed volume changes remain cumulative. At *P* > *P*_int_, compression of the liquids in the transitiometer proceeds slowly, which complicates separating the compressibility of bulk liquids from additional intrusion of water into the remaining empty or partially filled pores. The reported intruded water volume for the ITQ 1 sample is 0.14 cm^3^ g^–1^ (Figure 3).

In the present article, a specific procedure was applied to extract only the fast-changing component of the recorded *V*_exp_(*t*) (eq 1). The same method of data treatment was applied to the *V*(*P*) curve derived from porosimetry,^47^ by replacing *t* with *P* in eq 1. The results are shown in Figure S6. The calculated intruded water volume is 0.13 cm^3^ g^–1^, consistent with the 0.14 cm^3^ g^–1^ measured for the pristine ITQ 1 sample. This value also agrees with thermogravimetric (TG) analysis^47^: upon heating, the intruded MUL sample lost 12% of its mass, corresponding to an initial water content of approximately 0.14 cm^3^ g^–1^. Thus, the proposed method for decomposing raw *P*–*V* isotherms is believed to better reflect the underlying physics of water intrusion into porous materials.

The measured intruded volume is approximately half of the microporous volume of 0.25 cm^3^ g^–1^ determined by the Brunauer–Emmett–Teller (BET) method from N_2_ adsorption–desorption isotherms.^47^ This volume corresponds to one unit cell being occupied by approximately 20 water molecules, assuming the bulk water density is about 1 g/cm^3^.

Direct computer simulations of intrusion (Procedure 2) indicate that only a fraction of the supercells is filled when the applied pressure slightly exceeds *P*_int_. Intrusion initiates in cells near the surface of the cubic crystalline grain and subsequently propagates toward the crystal core (2 × 2 × 2 u.c.). Very slow penetration kinetics are observed (Figure S7), suggesting that thermodynamic equilibrium may not be fully reached on the simulation time scale. These slow kinetics hamper the direct determination of an intrusion isotherm.

An alternative interpretation is that, in the pressure range 20 < *P* < 100 MPa, a dynamic equilibrium exists between filled and empty cells. In this regime, the gradual increase in the intruded water volume can be rationalized by changes in the relative fraction of filled versus empty supercells rather than by continuous filling of individual cages. This penetration behavior is qualitatively different from that reported for channel-type materials.^34,36^

### 4.2 Heat of Intrusion

Heats of intrusion measured for LTA-type PSZs are shown in Figure 8. For both samples, the data were averaged at each temperature. The exothermic heat decreases with increasing temperature, in agreement with the simulations, and the experimental and calculated values are also in good agreement. Experimental uncertainties are substantial, particularly at elevated temperatures, where the released heat is small, and the transitiometer operates near its sensitivity limit (Figure S1). Nevertheless, the decrease in exothermic heat with temperature is clearly evident. A similar trend has been reported for ZIF-8, a material with cages.^31^ For comparison, all data were fitted with straight lines. Although the slopes differ, the specific heats of intrusion for both materials approach zero over a narrow temperature range.

**Figure 8:**
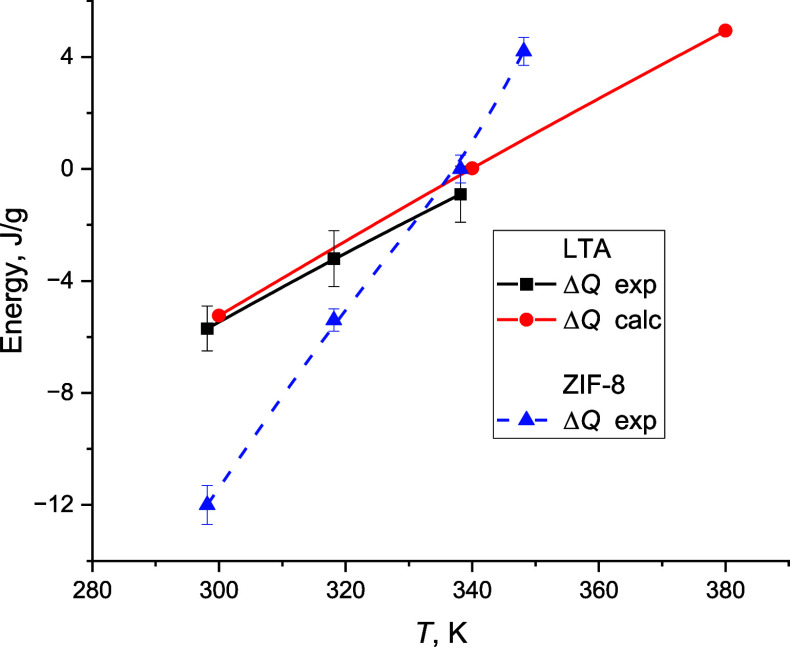
Temperature dependence of the heats of intrusion for LTA-type pure-silica zeolite and MOF ZIF-8.

Another factor contributing to the observed uncertainties is the gradual degradation of the material caused by the hydrolysis of silicate bonds. High-temperature treatment under low pressure only partially restores the structure of the LTA zeolite (MUL).^47,48^ The number of internal silanol groups increases with successive intrusion–extrusion cycles, enhancing the hydrophilicity of the framework. After water intrusion, fractured crystals and partial amorphization of the material are observed in SEM micrographs.^47^ Results of X-ray diffraction, surface area, and pore volume measurements, as well as thermogravimetric analysis for the ITQ sample collected before and after the transitiometer experiment and after high-temperature treatment, are provided in the SI (Figures S1–S3, Table S1). The number of silanol groups was estimated to be approximately 1.7 OH groups per unit cell (Si_2__4_O_4__8_) for the nonintruded sample and about 4.2–4.4 OH groups per unit cell for the samples subjected to the transitiometer experiments. The corresponding reduction in pore volume is about 15%. Consequently, the number of intact pores decreases with repeated intrusion–extrusion and recovery cycles. Together with the temperature-induced decrease in pore loading, this results in a gradual decline of the intruded volume per gram of material.

Heat effects were calculated under the assumption that all supercells are fully occupied by water (Procedure 1). In real crystals, however, some cages may remain empty, implying that the heat effects for pristine, defect-free materials could be underestimated. Conversely, the formation of silanol defects alters the crystal structure,^62,79^ reduces hydrophobicity, and is accompanied by additional heat effects associated with chemical reactions. Silanol groups interact more strongly with water than framework oxygen atoms and therefore can serve as nucleation centers for water intrusion.^43^

Despite these inherent limitations and uncertainties, the calculated results exhibit remarkably good agreement with experimental data. The proposed approach, therefore, provides a reliable framework for rationalizing and estimating the heat effects associated with water intrusion in porous materials.

We note that at ambient temperature, the heat effects have opposite signs for materials with cage-type and channel-type pores. Porous materials such as CHA-type PSZ and hydrophobized mesoporous silica gel EVA exhibit exothermic intrusion,^1,80^ with *Q* = −7.85 and −6.5 J g^–1^, respectively—values of the same order of magnitude as those for LTA and ZIF-8. In contrast, water intrusion into materials with channel-like pores, such as MFI zeolite, certain mesoporous silicas, and some MOFs, is endothermic.^56^

### 4.3 Water Properties

New ice structures have been reported for water confined within carbon nanotubes under high pressure.^81–84^ A characteristic feature of these phases is the formation of four-membered hydrogen-bonded rings near the tube surface. Similar structural motifs have also been observed for liquid water confined within hydrophobic nanotubes of square cross-section.^24^

Representative snapshots of water clusters in LTA cages are shown in Figure 9. These structures were obtained through energy minimization and correspond to local minima on the potential energy surface. At lower loading (*N*_*w*_ = 20), four-, five-, and six-membered rings—the principal structural motifs of liquid water^24^—are clearly visible. With increasing water density in the cage, the fraction of four-membered rings rises. At *N*_*w*_ = 30 water molecules remain mobile, yet fragments of quadratic-ice-like crystalline ordering are apparent. The reduced entropy reflects the loss of structural diversity, as molecules preferentially form four H bonds with their neighbors, which lowers potential energy and decreases the number of dangling O–H groups. At high loading, water exhibits increased ordering and significantly hindered mobility, consistent with a viscous state. However, this order is lost at 380 K. Movies S1 and S2 present a series of molecular configurations at high loading (*N*_*w*_ = 28), allowing frame-by-frame inspection of the water structure and the contrast in molecular mobility at 300 and 380 K.

**Figure 9:**
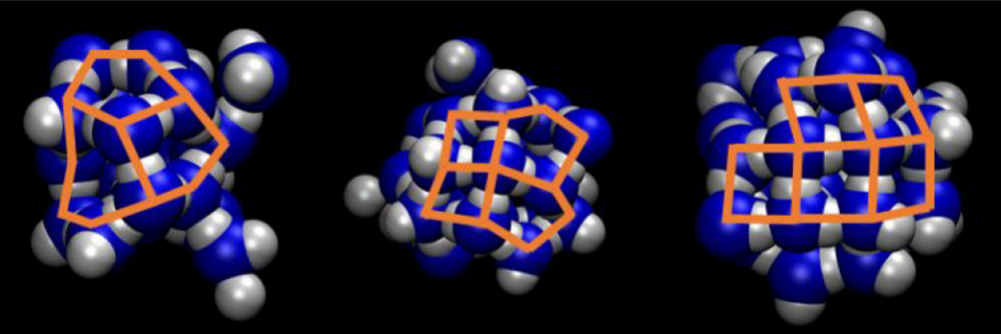
Representative snapshots of optimized water clusters in LTA-type zeolite supercells for *N*_*w*_ = 20 (left), 25 (middle), and 30 (right). Hydrogen bonds are shown as lines connecting selected oxygen atoms.

The evolution of the optimized water cluster structures upon loading is illustrated in Figure S8. Two competing tendencies govern the cluster morphology: the formation of chains and the formation of globules. For *N*_*w*_ = 4–6, chain-like structures connecting adjacent cages predominate. Movie S3 shows the dynamics of a water cluster consisting of six molecules at 300 K. Most of the time, the cluster remains globular, but rapid transformations into chain-like configurations are occasionally observed.

At higher loadings (*N*_*w*_ = 7–15), globular clusters become predominant. Initially, the clusters connect cages along one direction, and subsequently along two and three directions (Figure S8). Both the self-diffusion coefficient (Figure 7) and the number of broken hydrogen bonds (Figure 6c) reach a maximum in the range where globular water clusters begin to interconnect neighboring cages. In this regime, the water molecules have sufficient free volume and kinetic energy to hop between adjacent cages. At *N*_*w*_ > 14, all cages become interconnected, and water molecules occupy all 8MR windows. Upon further loading, the formation of dense globules is observed (Figure 9).

If individual water molecules or small clusters evaporate into an empty cage of LTA, they must overcome a substantial energetic barrier (Figure 10). Previous studies have shown that, in porous materials with interconnected channels, such connectivity facilitates water penetration by enabling the formation of hydrogen-bonded networks spanning adjacent channels.^33,34,36^ In LTA, the 8MR windows connect neighboring cages along three mutually perpendicular directions. Simulations performed according to Procedure 2 indicate that water molecules from the bulk phase initially occupy cages near the surface of the crystalline grain. To penetrate an empty cage in the grain core, small chain-like clusters form that connect the filled cages. These clusters are stabilized through hydrogen bonding with water molecules in the neighboring filled cages (Figure S9).

**Figure 10:**
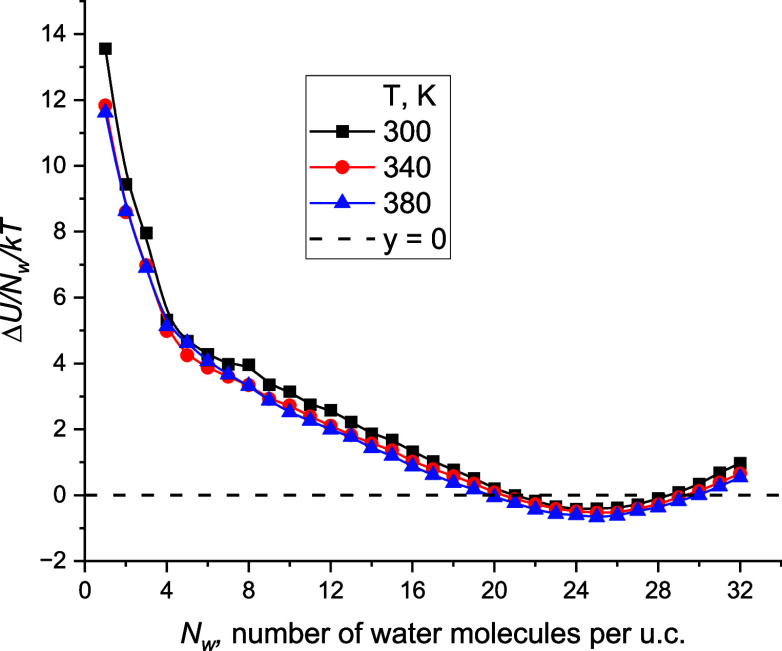
Excess internal energy of water molecules in LTA-type PSZ relative to bulk water, calculated per molecule in the unit cell and normalized by *k*_B_ *T*.

A snapshot illustrating an early stage of pore filling is shown in Figure 11, where, for clarity, only two core cages (green) and two surface cages (orange) of the 64-supercell grain are displayed. The chain of water molecules entering an empty cage is stabilized by H bonds with molecules in the upper, lower, and left neighboring cages. This snapshot represents only one possible configuration; additional examples are provided in Figure S10. The concerted motion of several molecules is energetically more favorable than the sequential condensation of individual molecules within a cage. Thus, the same stabilization mechanism operates in materials with cage-like pores as in those with channel-type structures.

**Figure 11:**
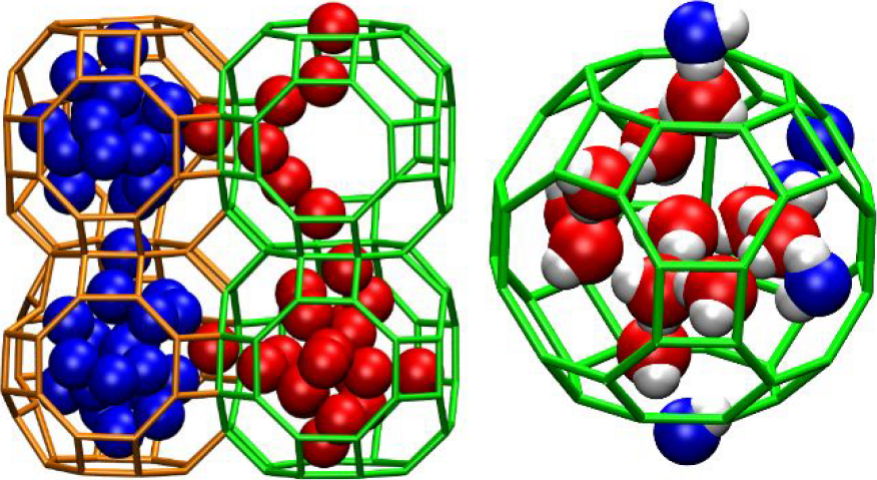
Water penetration into the LTA crystal at an early stage (left) and at the moment corresponding to the putative formation of a critical nucleus (right). Two core cages are outlined by green lines connecting silicon atoms of the zeolite framework. Orange lines connect silicon atoms in surface cages. Red spheres represent oxygen atoms of water molecules located in the core cages.

As shown in Figure 10, the energetic barrier for clusters containing 4–16 molecules is lower than that for smaller ones. The positive values of excess energy can be interpreted as a nucleation-like barrier for cage filling, but only in a qualitative sense. An intermediate cluster size (∼8 to 16 water molecules per cage), characterized by elevated potential energy and enhanced molecular mobility, is consistent with the notion of a critical nucleus under confinement.

Estimating the size of a critical nucleus for nanometer-scale clusters is a highly nontrivial task. Such clusters may have comparable probabilities of growth and dissolution, and even a supercritical water cluster can transiently disappear. Moreover, critical nuclei may differ in composition, shape, energy, and molecular connectivity, reflecting the heterogeneous nature of nucleation under nanoconfinement. Consequently, reliable statistics would require hundreds or thousands of independent nucleation events.^85^ This problem has been discussed previously in detail on the basis of molecular simulations of water penetration into ZIF-8 crystals.^27^

Figure S10 illustrates the formation (frames 102–109) and subsequent annihilation (frames 110–118) of a water cluster consisting of 11 molecules within an initially empty cage located in the core of the grain. The time interval between consecutive frames is 40 ps. We speculate that this cluster, also shown in Figure 11, corresponds to a putative critical nucleus, as it ultimately shrinks rather than grows, and similar clusters exhibit elevated excess potential energy and enhanced diffusion coefficients (Figures 5d and 7).

Classical nucleation theory is not applicable under such nanoconfinement conditions, as several of its fundamental assumptions are violated.^27^ In particular, it is not possible to clearly distinguish between two bulk-like water phases in Figures 11 and S10. Moreover, the size of the critical nucleus depends sensitively on the occupancy of neighboring cages and may be reduced when adjacent cages are fully filled. Frames 119–131 in Figure S10 show the formation and growth of another water cluster, which subsequently nucleates and evolves into the final structure observed in Figure S9.

Previously,^24^ for straight nanotubes with a square cross-section, the intrusion pressure was found to be proportional to the inverse pore aperture (*d* ^–1^), in accordance with the Laplace–Washburn equation, which defines the capillary pressure as

$$P_{c}=4\frac{\gamma_{\mathrm{sl}}-\gamma_{\mathrm{sv}}}{d}$$ 8

where γ_sl_, γ_sv_ are solid–liquid and solid–vapor surface tensions, respectively.

It is not possible to determine the pore aperture at the atomistic level in an unambiguous manner. While distances between atomic centers are well-defined, atoms are more appropriately represented as soft spheres, particularly in molecular dynamics simulations employing classical force fields. An additional complication arises from the complex geometry of zeolite pores, which often exhibit noncircular cross sections and irregular windows connecting adjacent cages (Figures 1 and S11).

In the present work, we therefore employ two structural descriptors: (*a*) the maximum diameter of a sphere able to diffuse through the pore system, taken from the Database of Zeolite Structures of the Structure Commission of the International Zeolite Association^37^; and (*b*) the ratio of Connolly accessible surface area to free volume, *A/V*, evaluated using a probe particle radius of 2 Å.^86,52,33^ For an ideal macroscopic cylindrical pore, the ratio of lateral surface area to volume scales as *d* ^–1^.

The intrusion pressures together with the topological and geometric characteristics of PSZs are summarized in Table S2. Experimental data were taken from the literature,^22^ and the methodology for surface area and volume calculations has been described previously.^33^ The Connolly accessible surface area and volume for LTA were calculated, accounting for the absence of water in the sodalite cages, while the intrusion pressure for LTA was obtained in the present work. As shown in Figure 12a, the intrusion pressures do not correlate directly with *d* ^–1^. A pronounced difference is observed between materials with 1D channel systems and those with cage-type pore architectures, which form distinct families.

**Figure 12:**
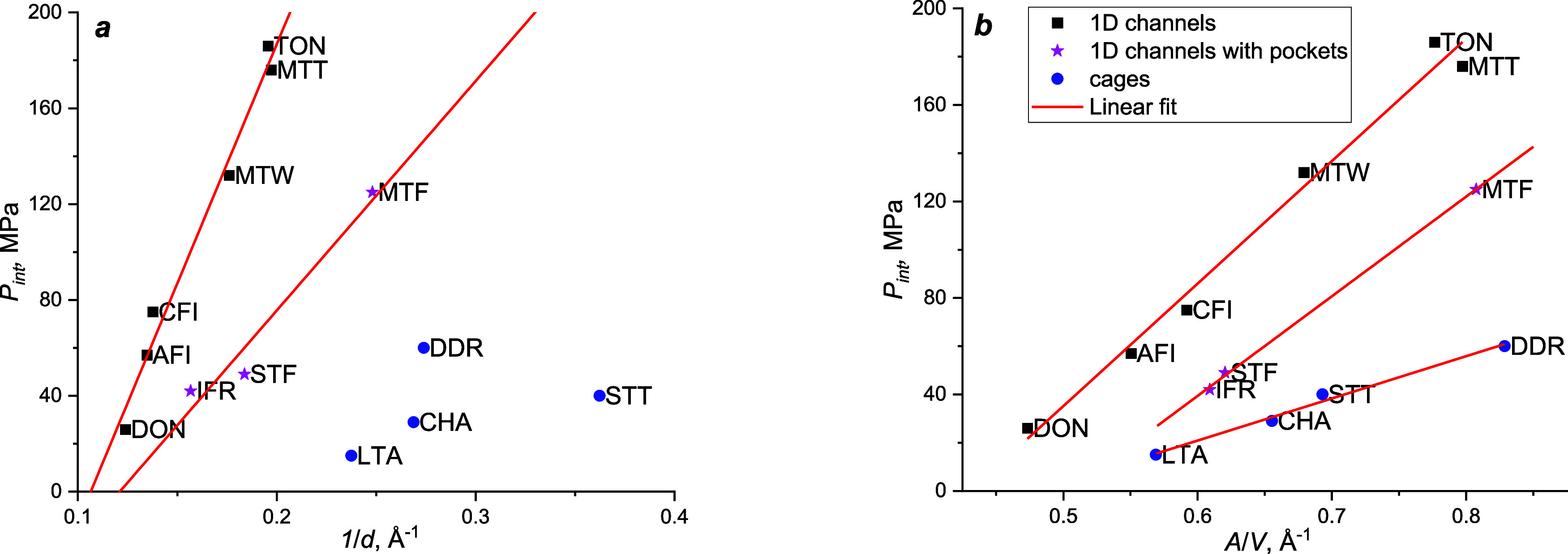
Correlation between intrusion pressure and structural descriptors of pure silica zeolites: (a) 1/*d*, where *d* is the maximum diameter of a sphere able to diffuse through the pore system; (b) the ratio of Connolly accessible surface area to free volume.

It was previously noted^33^ that the intrusion pressures of PSZs with IFR, STF, and MTF topologies deviate from the correlation line established for zeolites with 1D channels, despite being of the same material class. This deviation originates from the presence of side pockets—a structural motif combining channels and cages. Such a material can, for example, be derived from an LTA-type PSZ by closing the 8MR windows in two directions. According to the established mechanism of water intrusion, this structural modification is expected to increase the intrusion pressure. Similar behavior has been reported for ITT- and MFI-type PSZs,^34^ hydrophobic nanotubes,^33^ and hierarchical meso/microporous level-2 and level-3 Menger sponge particles.^36^ The MTF-type PSZ possesses the same 8MR windows as LTA; however, its intrusion pressure is approximately an order of magnitude higher. Even for STF and IFR PSZs, which contain larger pore windows between pockets (10MR and 12MR, respectively), the intrusion pressures remain several times higher than that of LTA.

Figure 12b exhibits a markedly stronger correlation than Figure 12a, particularly for zeolites with cage-type pore architectures. For zeolites with 1D channel systems, a linear dependence of the intrusion pressure on both structural descriptors is expected, since the surface-to-volume ratio scales as *d* ^–1^, even when the definition of *d* is somewhat ambiguous. Notably, for cage-based zeolites, Figure 12b also reveals an approximately linear correlation between *P*_*int*_ and the *A/V* ratio.

It should be noted that only a subset of the framework topologies reported in the IZA database has been realized in synthesized PSZs, and intrusion pressures have not been measured for all such materials. Nevertheless, the observed trends indicate that the intrusion pressure can be rationalized, and potentially estimated, on the basis of the pore architecture and the surface-to-volume ratio.

Previously published computer simulations of water intrusion into LTA and CHA zeolites (Figure 1), as well as MFI-type PSZs at 300 K, have revealed a strong similarity in the thermodynamic behavior of zeolites with cage-like pores, in contrast to those featuring three-dimensional channel systems such as MFI.^25^ Unlike materials with cage-like porosity (LTA, CHA, ZIF-8), PSZs with channel-type pores exhibit opposite heat effects, with water being energetically more stable within hydrophobic cages. At comparable surface area-to-pore volume ratios, the intrusion pressures are lower for materials with cage-like pores. Another key distinction between these two structural types is the sensitivity of intrusion pressure to the pressurization rate, which is more pronounced in cage-like systems.^27^ Nevertheless, the nature of pore connectivity plays a crucial role in both types of porous structures: interconnections between pores facilitate water intrusion and thus lower the intrusion pressure.

The excess interfacial free energies for confined water deviate substantially from solid–liquid and solid–vapor surface tensions in the Laplace–Washburn eq (eq 8) due to the effects of pore topology, shape, degree of interconnection, water loading, and molecular clustering under confinement—rather than solely because of the pore aperture size (*d*). Therefore, even when the chemical composition of the surface and the pore aperture size are identical, the intrusion pressures can differ significantly. These parameters are less critical than the internal surface area, the free pore volume, and the nature of pore connectivity.

## 5 Conclusions

For the first time, combined calorimetric measurements and molecular simulations provide detailed insights into the water intrusion process in hydrophobic LTA-type zeolites with cage-like pores at three temperatures. Pure-silica LTA samples synthesized by two distinct methods were investigated, and a scanning transitiometer was used to simultaneously record pressure–volume isotherms and differential heats.

A novel data-processing method, based on separating fast and slow volume changes during intrusion at a constant pressurization rate, was proposed and successfully applied. This approach revealed the dependence of intruded volume, intrusion pressure, and heat of intrusion on both the synthesis route and temperature.

Among the studied water–PSZ systems, LTA-type zeolite exhibited the lowest intrusion pressure (12–17 MPa), which is only weakly dependent on temperature. Water remained within the pores after pressure release, characterizing the system as a molecular bumper. Water intrusion in LTA-type PSZs is exothermic at 298 K, with an average heat of −5 J g^–1^. The degree of exothermic heat decreases with increasing temperature, approaching zero above 338 K, consistent with behavior reported for MOF ZIF-8, which also features cage-like pores. At ambient temperature, intrusion is exothermic for materials with cage-like pores but endothermic for those with channel-type pores.

Simulations demonstrate slow intrusion kinetics. At pressures slightly above the intrusion pressure, only a fraction of the pores within the core of the crystalline grain are occupied by water, establishing a dynamic equilibrium between empty and filled pores over a broad pressure range.

The most energetically stable states are observed for 22–28 molecules per LTA supercell. At high loadings, four-membered hydrogen-bond rings form at the surfaces of globular water clusters, resembling ice-like motifs observed for confined water in hydrophobic nanotubes under high pressure. The preferred occupancy of 17–22 molecules per supercell reflects the balance between competing entropic and energetic factors. Experimental and simulated heats are in close agreement, both predicting a transition from exothermic to endothermic behavior with increasing temperature.

The onset of empty-cell filling involves a collective, concerted movement of water molecules that facilitates overcoming the energetic barrier. Hydrogen-bond statistics indicate that the largest number of broken bonds relative to bulk water occurs when 8–16 molecules occupy the cage, corresponding to the highest excess energy and molecular mobility. The size of the critical nucleus formed within the cage depends on the loading of neighboring cages and is estimated to fall within this range.

The strong dependence of energetic characteristics on water loading indicates that the Laplace–Washburn equation, commonly used to predict intrusion pressures in capillaries, is not applicable to nanopores. For straight model nanotubes and grafted mesoporous silicas, linear correlations with the inverse pore diameter have been reported; however, such correlations fail for PSZs.

Notably, for cage- versus channel-type PSZs, distinct linear relationships emerge between intrusion pressure and the ratio of Connolly accessible surface area to free pore volume, reflecting the dominant influence of pore topology on water intrusion behavior. The key factors governing intrusion pressure in any PSZ are the geometry and connectivity of pores. The molecular mechanism responsible for energetic stabilization—and the resulting decrease in intrusion pressure—arises from the formation of hydrogen bonds between water molecules located within a pore and those in adjacent pores.

High energy output can be achieved during water expulsion from nanopores in materials exhibiting exothermic extrusion, such as grafted mesoporous silicas with straight, channel-type pores. The hydrophobicity of these materials—and consequently, their working pressure—can be tuned through chemical modification of the internal surface as well as adjustments in pore geometry and connectivity. Fast water jets generated during extrusion can propel micro- and nanoparticles, suggesting a potential principle for the design of nanoengines.

Overall, these findings provide a comprehensive understanding of water behavior in hydrophobic porous materials, emphasizing the critical roles of internal surface area, free pore volume, pore connectivity, and temperature in the intrusion–extrusion process. This knowledge enables the rational design of HLS-based systems for energy storage, mechanical actuation, and nanodevices, and establishes design principles for tailoring wetting and thermodynamic properties in next-generation porous frameworks.

## Supplementary Material













## Notes

Additional figures and results of zeolite characterization (PDF)
Water mobility in an LTA unit cell at 300 K with 28 water molecules (MPG)
Water mobility in an LTA unit cell at 380 K with 28 water molecules (MPG)
Water mobility in an LTA unit cell at 300 K with 6 water molecules (MPG)
DL_POLY input files for Procedure 2 in Methods (ZIP)
GULP input files for Procedure 1 in Methods (ZIP)

## References

[1] Coiffard L., Eroshenko V. A., Grolier J. P. E. (2005). Thermomechanics of the Variation
of Interfaces in Heterogeneous Lyophobic Systems. AIChE J..

[2] Fadeev A., Eroshenko V. (1997). Study of Penetration of Water into Hydrophobized Porous
Silicas. J. Colloid Interface Sci..

[3] Confalonieri G., Daou T. J., Nouali H., Arletti R., Ryzhikov A. (2020). Energetic
Performance of Pure Silica Zeolites under High-Pressure Intrusion
of LiCl Aqueous Solutions: An Overview. Molecules.

[4] Fraux G., Coudert F. X., Boutin A., Fuchs A. H. (2017). Forced Intrusion
of Water and Aqueous Solutions in Microporous Materials: From Fundamental
Thermodynamics to Energy Storage Devices. Chem.
Soc. Rev..

[5] Canivet J., Fateeva A., Guo Y., Coasne B., Farrusseng D. (2014). Water Adsorption
in MOFs: Fundamentals and Applications. Chem.
Soc. Rev..

[6] Bennett T. D., Coudert F. X., James S. L., Cooper A. I. (2021). The Changing State
of Porous Materials. Nat. Mater..

[7] Ryzhikov A., Daou T. J., Nouali H., Patarin J., Ouwehand J., Clerick S., De Canck E., Van Der Voort P., Martens J. A. (2018). Periodic Mesoporous Organosilicas as Porous Matrix
for Heterogeneous Lyophobic Systems. Microporous
Mesoporous Mater..

[8] Giacomello A., Casciola C. M., Grosu Y., Meloni S. (2021). Liquid Intrusion in
and Extrusion from Non-Wettable Nanopores for Technological Applications. Eur. Phys. J. B.

[9] Eroshenko V., Regis R. C., Soulard M., Patarin J. (2001). Energetics: A New Field
of Applications for Hydrophobic Zeolites. J.
Am. Chem. Soc..

[10] Hashemi-Tilehnoee M., Tsirin N., Stoudenets V., Bushuev Y. G., Chorążewski M., Li M., Li D., Leão J. B., Bleuel M., Zajdel P., Del Barrio E. P., Grosu Y. (2023). Liquid Piston Based on Molecular
Springs for Energy Storage Applications. J.
Energy Storage.

[11] Amayuelas E., Farrando-Perez J., Missyul A., Grosu Y., Silvestre-Albero J., Carrillo-Carrión C. (2024). Fluorinated Nanosized
Zeolitic-Imidazolate
Frameworks as Potential Devices for Mechanical Energy Storage. ACS Appl. Mater. Interfaces.

[12] Hughes Z. E., Carrington L. A., Raiteri P., Gale J. D. (2011). A Computational
Investigation into the Suitability of Purely Siliceous Zeolites as
Reverse Osmosis Membranes. J. Phys. Chem. C.

[13] Rangnekar N., Mittal N., Elyassi B., Caro J., Tsapatsis M. (2015). Zeolite Membranes
- a Review and Comparison with MOFs. Chem. Soc.
Rev..

[14] Gritti F., Brousmiche D., Gilar M., Walter T. H., Wyndham K. (2019). Kinetic Mechanism
of Water Dewetting from Hydrophobic Stationary Phases Utilized in
Liquid Chromatography. J. Chromatogr. A.

[15] El-Naggar K., Yang Y., Tian W., Zhang H., Sun H., Wang S. (2024). Metal-Organic Framework-Based
Micro-/Nanomotors for Wastewater Remediation. *Small*. Science..

[16] Liu X., Sun X., Peng Y., Wang Y., Xu D., Chen W., Wang W., Yan X., Ma X. (2022). Intrinsic Properties
Enabled Metal Organic Framework Micromotors for Highly Efficient Self-Propulsion
and Enhanced Antibacterial Therapy. ACS Nano.

[17] Liu C., Tian C., Guo J., Zhang X., Wu L., Zhu L., Du B. (2024). Research Progress
of Metal-Organic Frameworks as Drug
Delivery Systems. ACS Appl. Mater. Interfaces.

[18] Lei Q., Guo J., Noureddine A., Wang A., Wuttke S., Brinker C. J., Zhu W. (2020). Sol–Gel-Based Advanced Porous Silica Materials for Biomedical
Applications. Adv. Funct. Mater..

[19] Ruiz-González N., Esporrín-Ubieto D., Kim I. D., Wang J., Sánchez S. (2025). Micro- and
Nanomotors: Engineered Tools for Targeted
and Efficient Biomedicine. ACS Nano.

[20] Erdosy D. P., Wenny M. B., Cho J., DelRe C., Walter M. V., Jiménez-Ángeles F., Qiao B., Sanchez R., Peng Y., Polizzotti B. D., de la Cruz M. O., Mason J. A. (2022). Microporous Water with High Gas Solubilities. Nature.

[21] Grosu Y., Li M., Peng Y. L., Luo D., Li D., Faik A., Nedelec J. M., Grolier J. P. (2016). A Highly
Stable Nonhysteretic {Cu2­(Tebpz)
MOF+water} Molecular Spring. ChemPhysChem.

[22] Ronchi L., Patarin J., Nouali H., Daou T. J., Ryzhikov A. (2024). Structure
Influence on High-Pressure Water Intrusion in Pure Silica Zeolites. New J. Chem..

[23] Laouir A., Luo L., Tondeur D., Cachot T., Le Goff P. (2003). Thermal Machines Based
on Surface Energy of Wetting: Thermodynamic Analysis. AIChE J..

[24] Bushuev Y. G., Grosu Y., Chorążewski M. (2024). Spontaneous Dipole
Reorientation in Confined Water and Its Effect on Wetting/Dewetting
of Hydrophobic Nanopores. ACS Appl. Mater. Interfaces.

[25] Lowe A. R., Chorażewski M. A., Grosu Y., Bushuev Y. G. (2024). Energetic
Characteristics of Hydrophobic Porous Materials as Candidates for
Manufacturing of Nanorockets ˙. J. Phys.
Chem. Lett..

[26] Wu J. K., Li J. H., Gu X. F., Huang J., Xu H. F., Wang C., Wang L., Liang J. G. (2024). Nanomotors Driven
by Waves with Different Frequencies. Nano Mater.
Sci..

[27] Sun Y., Rogge S. M. J., Lamaire A., Vandenbrande S., Wieme J., Siviour C. R., Van Speybroeck V., Tan J. C. (2021). High-Rate Nanofluidic Energy Absorption in Porous Zeolitic
Frameworks. Nat. Mater..

[28] Karbowiak T., Paulin C., Bellat J. P. (2010). Determination
of Water Intrusion
Heat in Hydrophobic Microporous Materials by High Pressure Calorimetry. Microporous Mesoporous Mater..

[29] Ryzhikov A., Dirand C., Astafan A., Nouali H., Daou T. J., Bezverkhyy I., Chaplais G., Bellat J. P. (2024). Calorimetric Heats
of Intrusion of LiCl Aqueous Solutions in Hydrophobic MFI-Type Zeosil:
Influence of the Concentration. Langmuir.

[30] Karbowiak T., Weber G., Bellat J. (2014). Geometry on the Energy of Intrusion. Langmuir.

[31] Lowe A. R., Ślęczkowski P., Arkan E., Le Donne A., Bartolomé L., Amayuelas E., Zajdel P., Chorążewski M., Meloni S., Grosu Y. (2024). Exploring the Heat of Water Intrusion
into a Metal-Organic Framework by Experiment and Simulation. ACS Appl. Mater. Interfaces.

[32] Bartolomé L., Anagnostopoulos A., Lowe A. R., Ślęczkowski P., Amayuelas E., Le Donne A., Wasiak M., Chorażewski M., Meloni S., Grosu Y. (2024). Tuning Wetting-Dewetting Thermomechanical
Energy for Hydrophobic Nanopores via Preferential Intrusion. J. Phys. Chem. Lett..

[33] Bushuev Y. G., Grosu Y., Chorążewski M. A., Meloni S. (2022). Effect of
the Topology on Wetting and Drying of Hydrophobic Porous Materials. ACS Appl. Mater. Interfaces.

[34] Bushuev Y. G., Grosu Y., Chorążewski M. A., Meloni S. (2022). Subnanometer
Topological Tuning of the Liquid Intrusion/Extrusion Characteristics
of Hydrophobic Micropores. Nano Lett..

[35] Wu L., Li Y., Fu Z., Su B. L. (2020). Hierarchically Structured Porous
Materials: Synthesis Strategies and Applications in Energy Storage. Natl. Sci. Rev..

[36] Bushuev Y. G. (2025). Effects
of Size and Porosity on the Hydrophobicity of Hierarchical Nanoparticles. Nano Lett..

[37] Baerlocher, C. ; McCusker, L. B. ; Brouwer, D. ; Marler, B. ; Database of Zeolite Structures; Structure Commission of the International Zeolite Association, http://www.iza-structure.org/databases/ (accessed 2026–01–14).

[38] Collins F., Rozhkovskaya A., Outram J. G., Millar G. J. (2020). A Critical Review
of Waste Resources, Synthesis, and Applications for Zeolite LTA. Microporous Mesoporous Mater..

[39] Corma A., Rey F., Rius J., Sabater M. J., Valencia S. (2004). Supramolecular Self-Assembled
Molecules as Organic Directing Agent for Synthesis of Zeolites. Nature.

[40] Gribov E. N., Sastre G., Corma A. (2005). Influence
of Pore Dimension and Sorption
Configuration on the Heat of Sorption of Hexane on Monodimensional
Siliceous Zeolites. J. Phys. Chem. B.

[41] Morris B. (1968). Heats of Sorption
in the Crystalline Linde-A Zeolite-Water Vapor System. J. Colloid Interface Sci..

[42] Faux D. A. (1999). Molecular
Dynamics Studies of Hydrated Zeolite 4A. J.
Phys. Chem. B.

[43] Randrianandraina J., Badawi M., Cardey B., Grivet M., Groetz J. E., Ramseyer C., Anzola F. T., Chambelland C., Ducret D. (2021). Adsorption of Water in Na-LTA Zeolites:
An Ab Initio
Molecular Dynamics Investigation. Phys. Chem.
Chem. Phys..

[44] Turgman-Cohen S., Araque J. C., Hoek E. M. V., Escobedo F. A. (2013). Molecular Dynamics
of Equilibrium and Pressure-Driven Transport Properties of Water through
LTA-Type Zeolites. Langmuir.

[45] Gorbach A., Stegmaier M., Eigenberger G. (2004). Measurement and Modeling of Water
Vapor Adsorption on Zeolite 4A - Equilibria and Kinetics. Adsorption.

[46] Castillo J. M., Silvestre-Albero J., Rodriguez-Reinoso F., Vlugt T. J. H., Calero S. (2013). Water Adsorption
in Hydrophilic Zeolites: Experiment and Simulation. Phys. Chem. Chem. Phys..

[47] Ryzhikov A., Ronchi L., Nouali H., Daou T. J., Paillaud J. L., Patarin J. (2015). High-Pressure Intrusion-Extrusion
of Water and Electrolyte
Solutions in Pure-Silica LTA Zeolite. J. Phys.
Chem. C.

[48] Confalonieri G., Ryzhikov A., Arletti R., Quartieri S., Vezzalini G., Isaac C., Paillaud J. L., Nouali H., Daou T. J. (2020). Structural Interpretation of the Energetic Performances
of a Pure Silica LTA-Type Zeolite. Phys. Chem.
Chem. Phys..

[49] Coudert F. X., Vuilleumier R., Boutin A. (2006). Dipole Moment, Hydrogen Bonding and
IR Spectrum of Confined Water. ChemPhysChem.

[50] Misturini A., Rey F., Sastre G. (2022). Molecular Simulation of Biobutanol Recovery Using LTA
and CHA Zeolite Nanosheets with an External Surface. J. Phys. Chem. C.

[51] Coudert F. X., Cailliez F., Vuilleumier R., Fuchs A. H., Boutin A. (2009). Water Nanodroplets
Confined in Zeolite Pores. Faraday Discuss..

[52] Bushuev Y. G., Sastre G., De Julián-Ortiz J. V., Gálvez J. (2012). Water-Hydrophobic
Zeolite Systems. J. Phys. Chem. C.

[53] Bushuev Y.
G., Sastre G. (2011). Atomistic
Simulation of Water Intrusion-Extrusion in
ITQ-4 (IFR) and ZSM-22 (TON): The Role of Silanol Defects. J. Phys. Chem. C.

[54] Van
der Perre S., Gelin P., Claessens B., Martin-Calvo A., Cousin Saint Remi J., Duerinck T., Baron G. V., Palomino M., Sánchez L. Y., Valencia S., Shang J., Singh R., Webley P. A., Rey F., Denayer J. F. M. (2017). Intensified
Biobutanol Recovery by Using Zeolites with Complementary Selectivity. ChemSusChem.

[55] Bouizi Y., Paillaud J. L., Simon L., Valtchev V. (2007). Seeded Synthesis of
Very High Silica Zeolite A. Chem. Mater..

[56] Lowe A. R., Wong W. S. Y., Tsyrin N., Chorążewski M. A., Zaki A., Geppert-Rybczyńska M., Stoudenets V., Tricoli A., Faik A., Grosu Y. (2021). The Effect
of Surface
Entropy on the Heat of Non-Wetting Liquid Intrusion into Nanopores. Langmuir.

[57] Sastre G., Gale J. D. (2005). Derivation of an
Interatomic Potential for Fluoride-Containing
Microporous Silicates and Germanates. Chem.
Mater..

[58] Combariza A. F., Gomez D. A., Sastre G. (2013). Simulating the Properties of Small
Pore Silica Zeolites Using Interatomic Potentials. Chem. Soc. Rev..

[59] Bai P., Tsapatsis M., Siepmann J. I. (2013). TraPPE-Zeo: Transferable Potentials
for Phase Equilibria Force Field for All-Silica Zeolites. J. Phys. Chem. C.

[60] Sastre G., Corma A. (2006). Rings and Strain in
Pure Silica Zeolites. J.
Phys. Chem. B.

[61] Bushuev Y. G., Sastre G. (2010). Atomistic Simulations
of Water and Organic Templates
Occluded during the Synthesis of Zeolites. Microporous
Mesoporous Mater..

[62] Bushuev Y. G., Sastre G. (2009). Atomistic Simulations
of Structural Defects and Water
Occluded in SSZ-74 Zeolite. J. Phys. Chem. C.

[63] Bushuev Y.
G., Sastre G. (2010). Feasibility
of Pure Silica Zeolites. J. Phys. Chem. C.

[64] Wu Y., Tepper H. L., Voth G. A. (2006). Flexible
Simple Point-Charge Water
Model with Improved Liquid-State Properties. J. Chem. Phys..

[65] Gale J. D. (1997). GULP: A
Computer Program for the Symmetry-Adapted Simulation of Solids. J. Chem. Soc. Faraday Trans..

[66] Gale J. D., Rohl A. L. (2003). The General Utility
Lattice Program (GULP). Mol. Simul..

[67] Todorov I. T., Smith W., Trachenko K., Dove M. T. (2006). DL_POLY_3: New Dimensions
in Molecular Dynamics Simulations via Massive Parallelism. J. Mater. Chem..

[68] Randzio S. L. (2007). Scanning
Transitiometry and Its Applications. J. Therm.
Anal. Calorim..

[69] Lowe A. R., Wong W. S. Y., Tsyrin N., Chorążewski M. A., Zaki A., Geppert-Rybczyńska M., Stoudenets V., Tricoli A., Faik A., Grosu Y. (2021). The Effect of Surface
Entropy on the Heat of Non-Wetting Liquid Intrusion into Nanopores
˙. Langmuir.

[70] Wadsö L. (2010). Operational
Issues in Isothermal Calorimetry. Cem. Concr.
Res..

[71] Snurr R. Q., Bell A. T., Theodorou D. N. (1993). Prediction
of Adsorption of Aromatic
Hydrocarbons in Silicalite from Grand Canonical Monte Carlo Simulations
with Biased Insertions. J. Phys. Chem..

[72] Persson R. A. X., Pattni V., Singh A., Kast S. M., Heyden M. (2017). Signatures
of Solvation Thermodynamics in Spectra of Intermolecular Vibrations. J. Chem. Theory Comput..

[73] Boddenberg B., Rakhmatkariev G. U., Hufnagel S., Salimov Z. (2002). A Calorimetric and
Statistical Mechanics Study of Water Adsorption in Zeolite NaY. Phys. Chem. Chem. Phys..

[74] Li J., Gao M., Yan W., Yu J. (2023). Regulation of the Si/Al Ratios and
Al Distributions of Zeolites and Their Impact on Properties. Chem. Sci..

[75] Perez-Carbajo J., Balestra S. R. G., Calero S., Merkling P. J. (2020). Effect of Lattice
Shrinking on the Migration of Water within Zeolite LTA. Microporous Mesoporous Mater..

[76] Kumar R., Schmidt J. R., Skinner J. L. (2007). Hydrogen
Bonding Definitions and
Dynamics in Liquid Water. J. Chem. Phys..

[77] Bukowski B. C., Keil F. J., Ravikovitch P. I., Sastre G., Snurr R. Q., Coppens M. O. (2021). Connecting Theory
and Simulation with Experiment for
the Study of Diffusion in Nanoporous Solids. Adsorption.

[78] Xiao H., Liang X. F., Zhou W., Jiang H., Parsons D. S., Yin H., Lu B., Sun Y. (2025). Stable Compressible Liquids Made
of Hierarchical MOF Nanocrystals. ACS Appl.
Mater. Interfaces.

[79] Bushuev Y. G., Sastre G., De Julián-Ortiz J. V. (2010). The Structural
Directing
Role of Water and Hydroxyl Groups in the Synthesis of Beta Zeolite
Polymorphs. J. Phys. Chem. C.

[80] Karbowiak T., Weber G., Bellat J. P. (2014). Confinement
of Water in Hydrophobic
Nanopores: Effect of the Geometry on the Energy of Intrusion. Langmuir.

[81] Liu Y., Jiang J., Pu Y., Francisco J. S., Zeng X. C. (2023). Evidence of Formation of 1 – 10 Nm Diameter
Ice Nanotubes in Double-Walled Carbon Nanotube Capillaries. ACS Nano.

[82] Mochizuki K., Adachi Y., Koga K. (2024). Close-Packed
Ices in Nanopores. ACS Nano.

[83] Wu Y., Wang Z., Li S., Su J. (2024). Terahertz Electric
Field Induced Melting and Transport of Monolayer Water Confined in
Double-Walled Carbon Nanotubes. Phys. Chem.
Chem. Phys..

[84] Trushin M., Andreeva D. V., Peeters F. M., Novoselov K. S. (2025). Structure
and Flow of Low-Dimensional Water. Nat. Rev.
Phys..

[85] Bushuev Y. G., Bartell L. S. (2007). Molecular Dynamics
Investigation of the Transient Regime
in the Freezing of Salt Clusters. J. Phys. Chem.
B.

[86] Connolly M. L. (1993). The Molecular
Surface Package. J. Mol. Graph..

