# Supplementary material for: Energetic and Structural Insights into Water Confined in Hydrophobic Nanopores
Source: J Phys Chem C Nanomater Interfaces. 2026 Jan 22;130(5):2057–71. doi: 10.1021/acs.jpcc.5c08036 (PMC13308698; doi:10.1021/acs.jpcc.5c08036)
Supplement: Supplementary file 1 [file jp5c08036_si_001.pdf]

SUPPORTING INFORMATION

Energetic and Structural Insights into Water Confined in Hydrophobic Nanopores

Yuriy G. Bushuev,[a*] *Alexander R. Lowe,* [a*] *Andrey Ryzhikov,* [b] *Tomasz Wasiak,* [a]

Michael Burt, [c,d] *Mirosław Chorążewski,* [a] *Yaroslav Grosu,*[a,e]

[a] *Institute of Chemistry, University of Silesia in Katowice, 40-006 Katowice, Poland*
[b] *Institut de Science des Matériaux de Mulhouse (IS2M), UMR 7361 CNRS, Axe Matériaux à Porosité Contrôlée (MPC), Université de Haute-Alsace, F-68100 Mulhouse, France; Université de Strasbourg, F-67000, Strasbourg, France*
[c] *The Chemistry Research Laboratory, Department of Chemistry, University of Oxford, Oxford, OX1 3TA, UK,*
[d] *Department of Chemistry, Trent University, Peterborough, Ontario, K9L 0G2, Canada*
[e] Centre for Cooperative Research on Alternative Energies (CIC energiGUNE), Basque Research and Technology Alliance (BRTA), Alava Technology Park, Albert Einstein 48, 01510 Vitoria-Gasteiz, Spain

Email: yuriy.bushuev@us.edu.pl; alexander.lowe@us.edu.pl

Characterization of Materials

X-ray Diffraction

X-ray diffraction patterns were collected on a PANalytical MPD X'Pert Pro diffractometer operating with Cu Kα radiation equipped with an X'Celerator detector at ambient temperature. The diffractogram of the ITQ sample heated at 330 °C for 2 h is characteristic of pure silica LTA-type zeolite. An increase in the amorphous phase content is observed for samples recovered after the transitiometer experiments. After subsequent heating at 330 °C, the fraction of the amorphous phase appears to decrease. The diffraction peaks of the samples after the transitiometer tests are slightly shifted toward higher 2θ angles, corresponding to a small contraction of the unit cell. Variations in the relative peak intensities are also observed between the samples before and after the transitiometer measurements. This effect is particularly pronounced for the first peak, (200) at 7.53° 2θ, whose relative intensity markedly decreases compared to the other reflections after the transitiometer experiments. This behavior can be ascribed to the formation of silanol groups and an increased water content within the pores. A similar effect was reported in previous study on MUL PSZs (Ryzhikov *et al.*, *J. Phys. Chem. C* **2015**, *119*, 28319). It should be noted that all diffractograms in Figure S4 were normalized to the intensity of the second peak, (220).

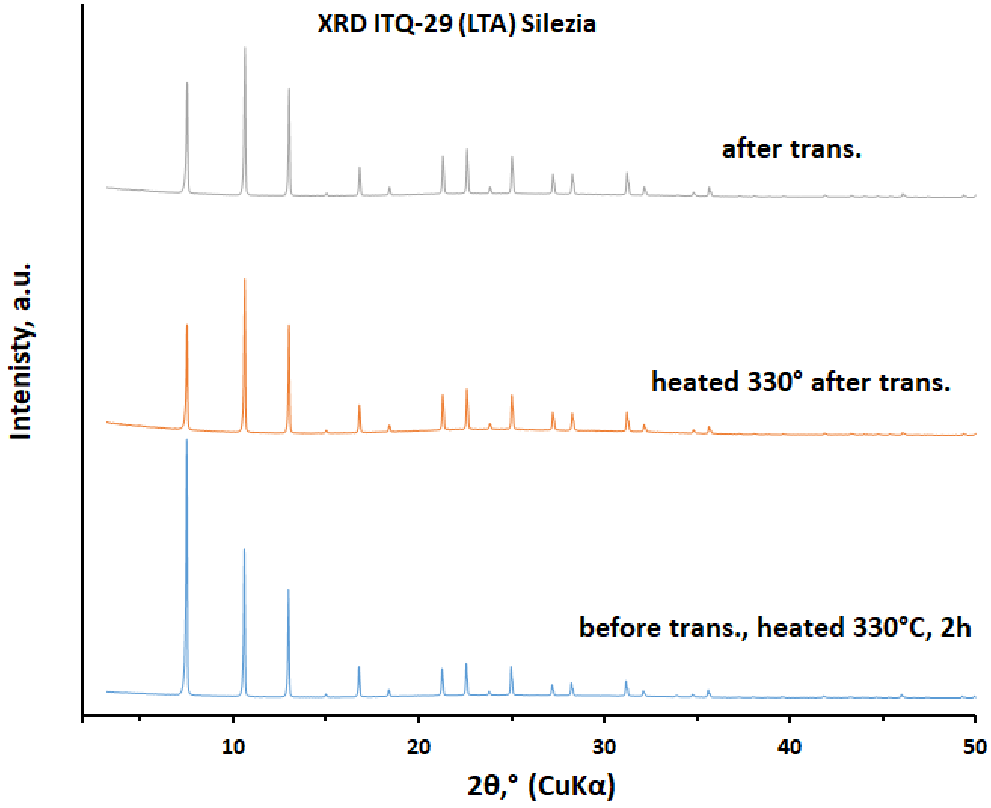

Figure S1. X-ray diffraction patterns of ITQ zeosil samples: before the transitiometer experiment, after the experiment, and after subsequent heating at 330 °C.

Textural Properties

Nitrogen adsorption−desorption isotherms were performed at 77 K using a Micromeritics ASAP 2420 apparatus. Before the measurements, the samples were outgassed at 90 °C overnight to eliminate physisorbed water, but not to impact the presence of silanol groups. The specific surface area and microporous volume were calculated using the BET and *t*-plot methods, respectively.

The textural parameters (specific surface area and pore volume) of the initial zeolite sample heated at 330 °C are typical of LTA-type zeosils. A decrease in both pore volume and specific surface area is observed after the transitiometer experiments. Subsequent heating of the post-transitometer sample at 330 °C results in a slight recovery of the textural properties. These results are consistent with previous findings for MUL (Ryzhikov *et al.*, *J. Phys. Chem. C* **2015**, *119*, 28319).

Table S1. Specific surface area, S_{BET}, and pore volume for the ITQ sample.

Sample	S_{BET}, m^2/g	V_{pore}, cm^3/g
ITQ heated at 330°C 2h	843	0.32
ITQ after transitiometer	695	0.26
ITQ after transitiometer, heated at 330°C	732	0.27

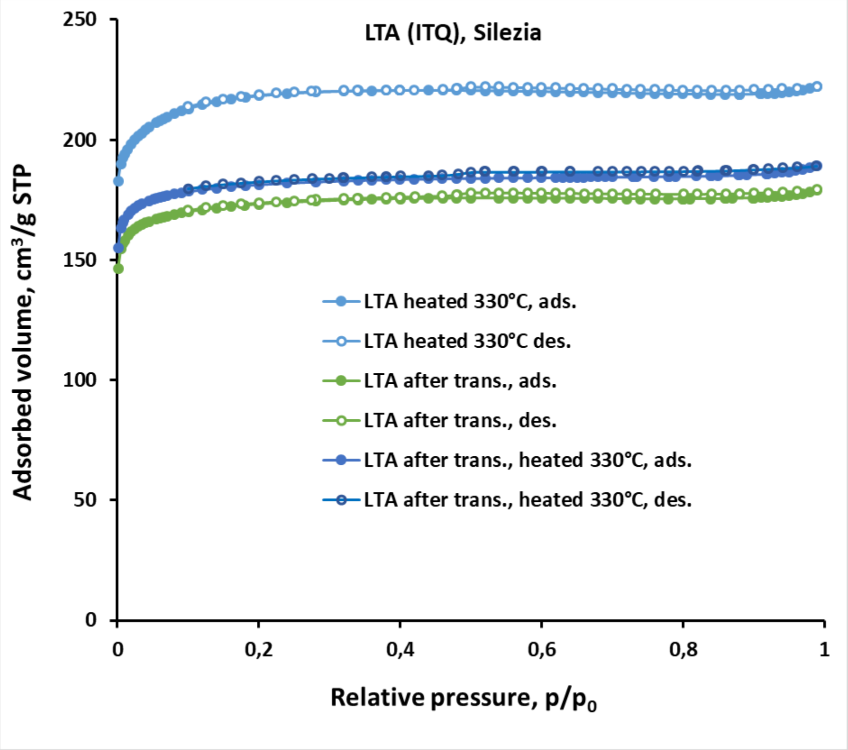

Figure S2. N_2 adsorption–desorption isotherms at −196 °C for ITQ zeosil samples: before the transitiometer experiment, after the experiment, and after subsequent heating at 330 °C.

Thermogravimetric Analysis

Thermogravimetric (TG) analyses were performed using a Mettler Toledo STARe apparatus, under air flow, at a heating rate of 5 °C/min in the range from 30 to 800 °C. Before the measurements, all the samples were stored for at least 24 hours under RH = 80 % in order to saturate them with water and to compare them in the same conditions.

The total weight loss of the ITQ sample prior to the transitiometer experiments (heated at 330 °C for 2 h) is approximately 3.3 wt%**,** which is higher than the value reported previously for MUL (1.7 wt%) (*Ryzhikov et al.*, *J. Phys. Chem. C* **2015**, *119*, 28319). Thus, the ITQ sample remains hydrophobic, though slightly less so than the MUL sample.

After the intrusion–extrusion experiments performed in the transitiometer, the thermogravimetric curve of the ITQ changes markedly. The total weight loss reaches 15.9 wt% (compared to 12.6 wt% for MUL). The first weight-loss step of about 13 wt%, observed between 30 and 300 °C, corresponds to the desorption of weakly and strongly physisorbed water molecules. The second weight-loss step above 300 °C is likely associated with the removal of water molecules produced during the condensation of silanol groups.

Based on this weight loss, which corresponds to the dihydroxylation reaction, the number of silanol groups was estimated to be ~1.7 OH groups per unit cell ($Si_{24}O_{48}$) for the non-intruded sample and ~4.2–4.4 OH groups per unit cell for the samples after the transitiometer experiments. No significant changes in the TG curves are observed after heating the post-transitiometer samples again at 330 °C.

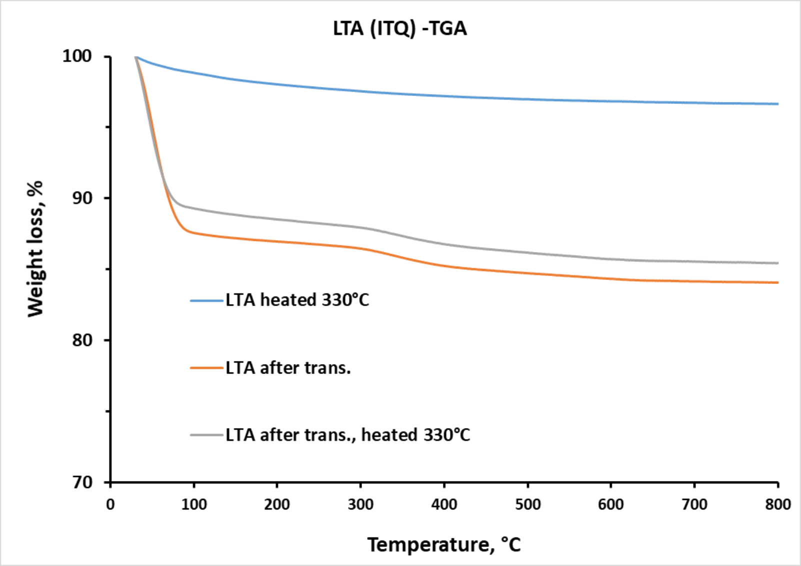

Figure S3. Thermogravimetric curves of ITQ samples: before the transitiometer experiment, after the experiment, and after subsequent heating at 330 °C.

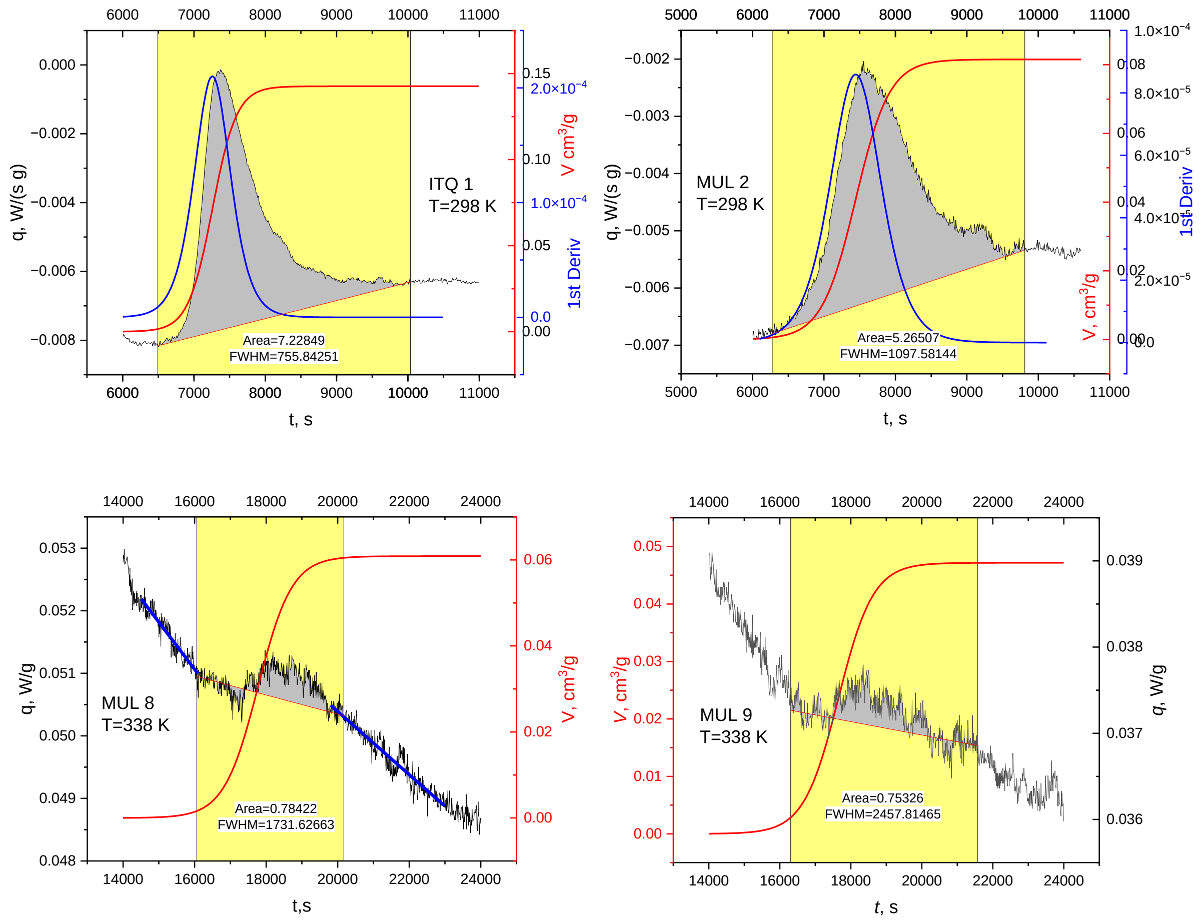

Figure S4. Evolution of the specific thermal power (q, black), intruded volume (V, red), and its derivative (dV/dt, blue) for ITQ-29 samples measured at 298.15 and 338.15 K.

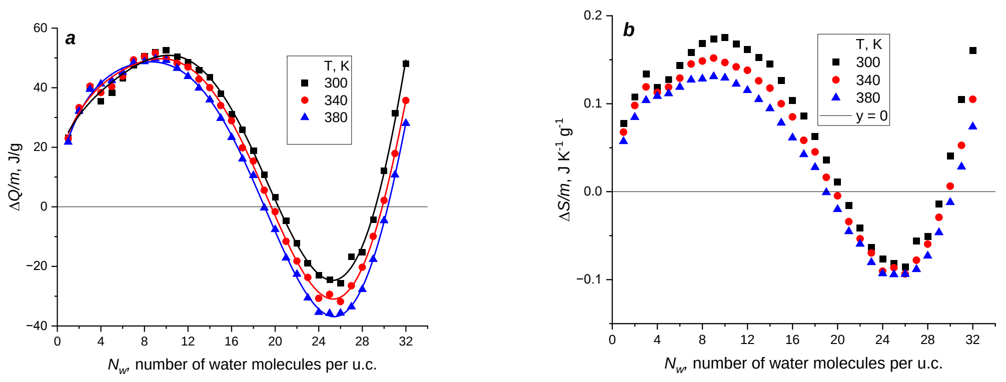

Figure S5. Thermodynamics of water intrusion into LTA: (a) heats of intrusion; (b) entropies for water transfer from bulk water into the zeolite.

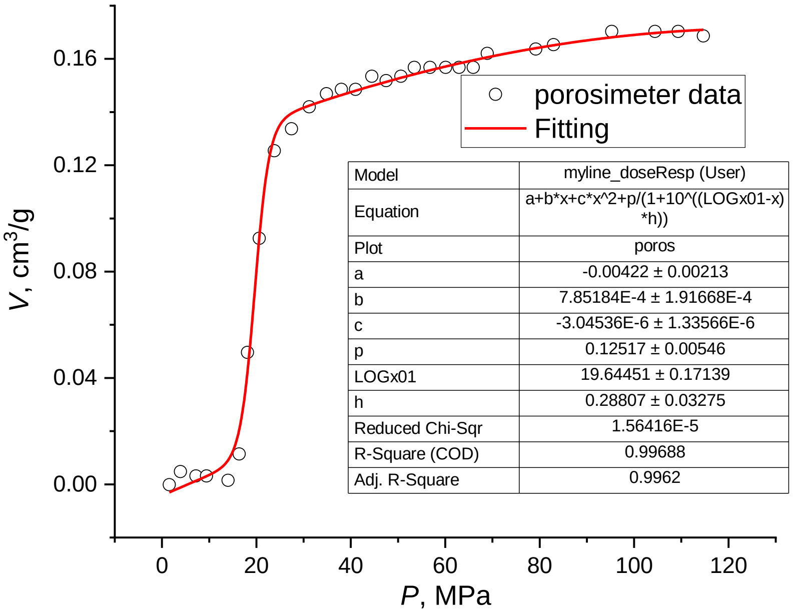

Figure S6. Water intrusion isotherm of LTA-type pure-silica zeolite (MUL) obtained using a high-pressure porosimeter, along with the fit of the experimental data according to eq 1.

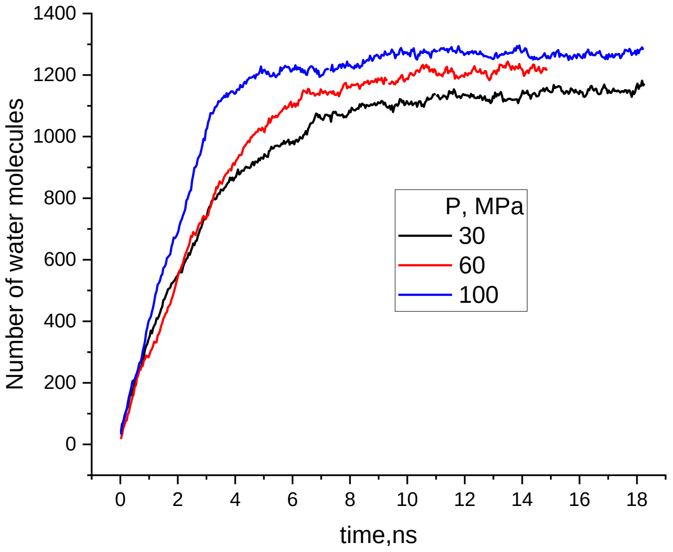

Figure S7. Time evolution of the number of water molecules intruding into the crystalline grain of LTA-type pure-silica zeolite containing 64 unit cells at different pressures.

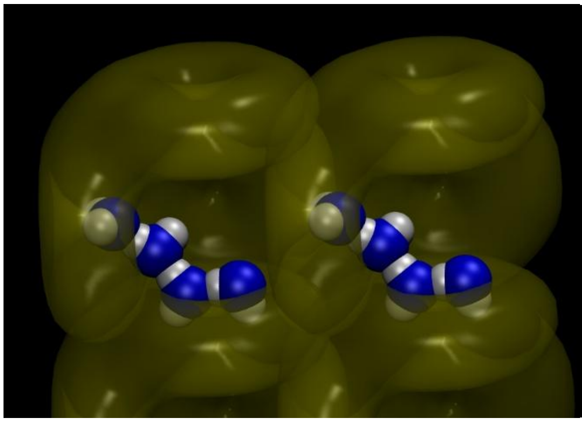

N_w=4

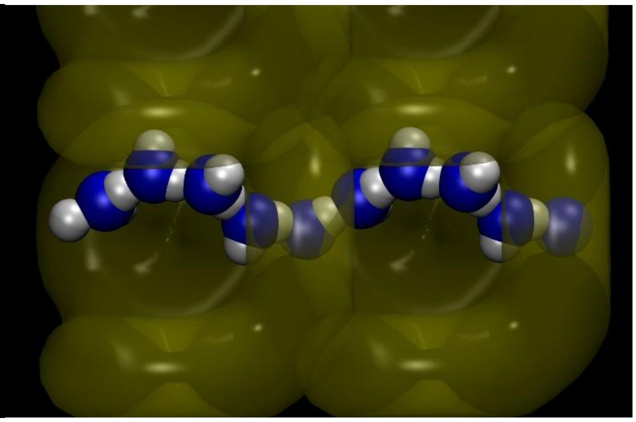

N_w =5

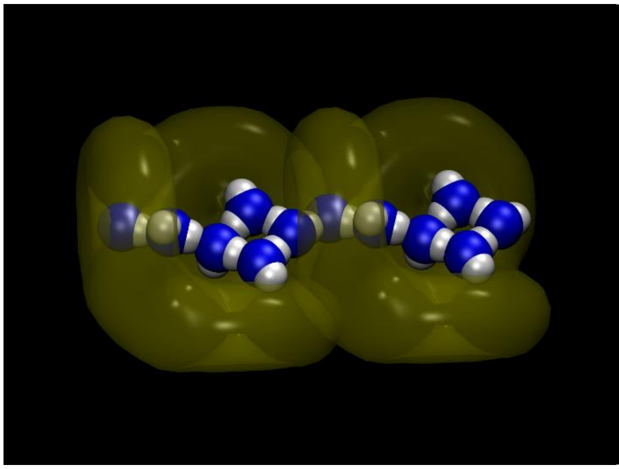

N_w=6

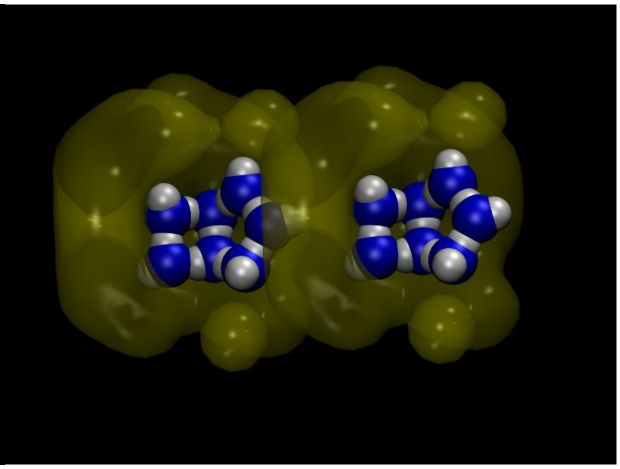

N_w =7

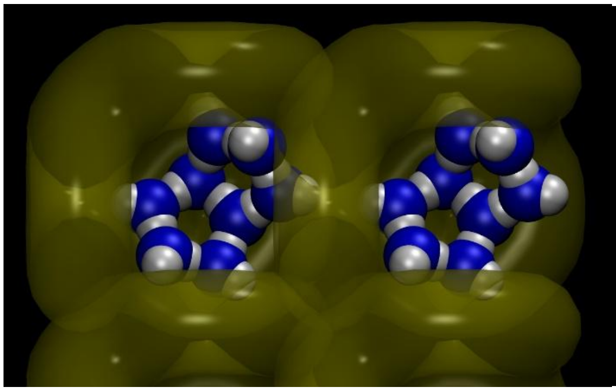

N_w=8

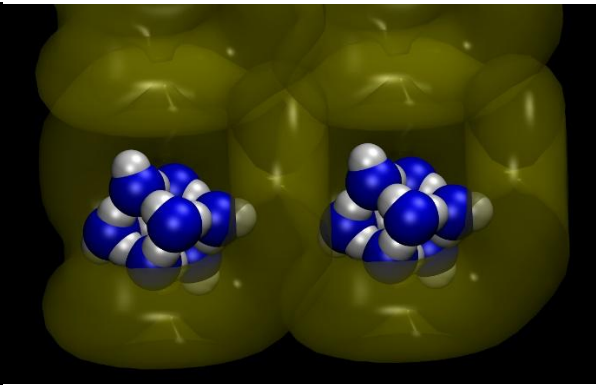

N_w =9

Figure S8. Optimized structures of water clusters in LTA as a function of loading (N_w). The configurations were obtained according to Procedure 1 and subsequently energy-minimized. All α-cages are equivalent. The LTA framework is represented as a transparent surface.

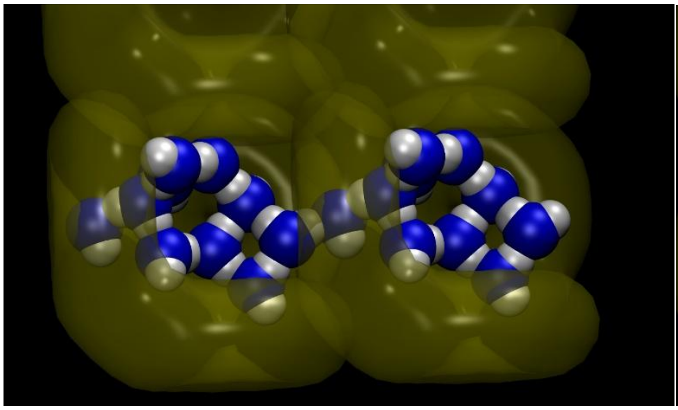

N_w=10

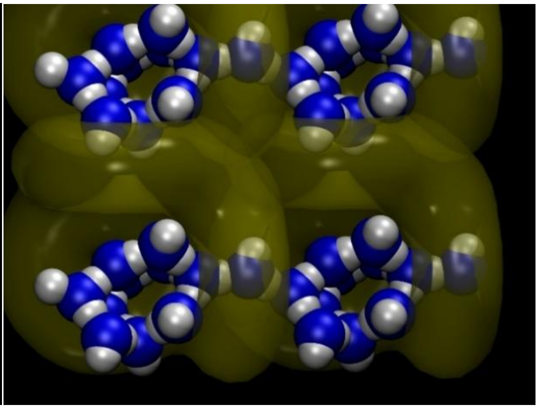

N_w =11

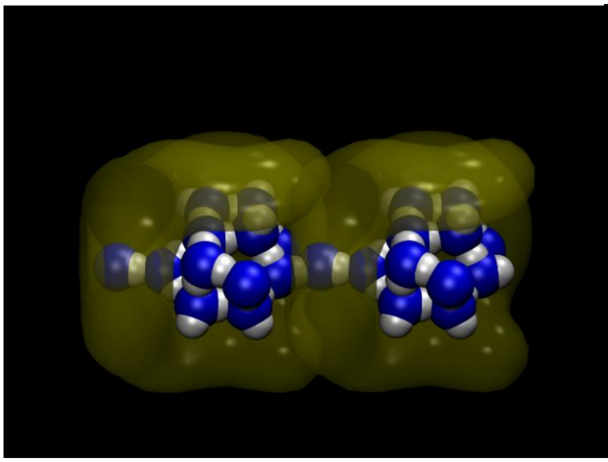

N_w=12

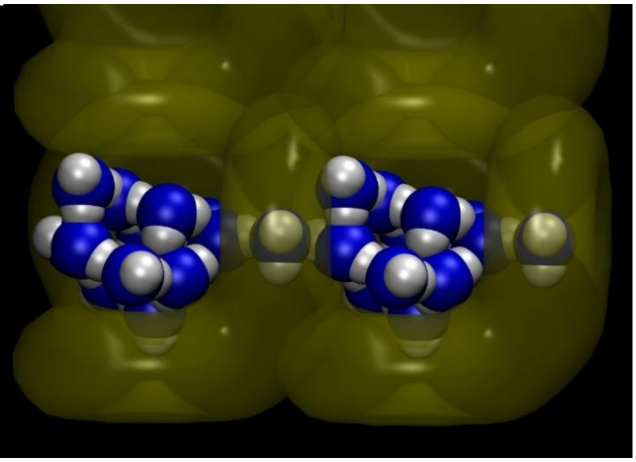

N_w =13

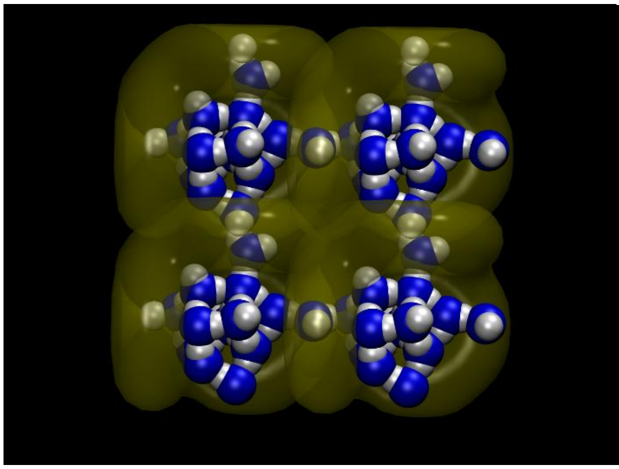

N_w=14

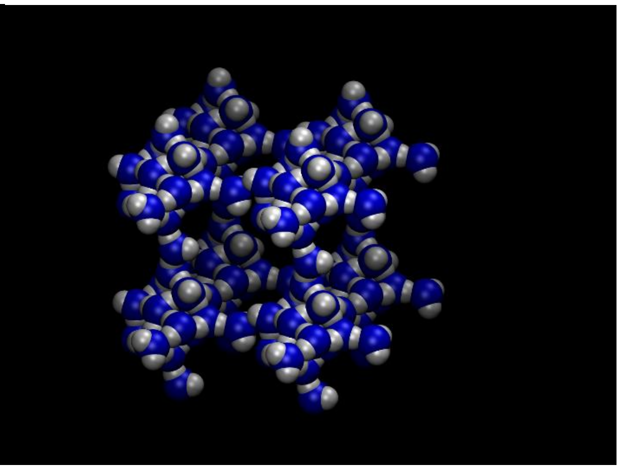

N_w =15

Figure S8. *Continued.*

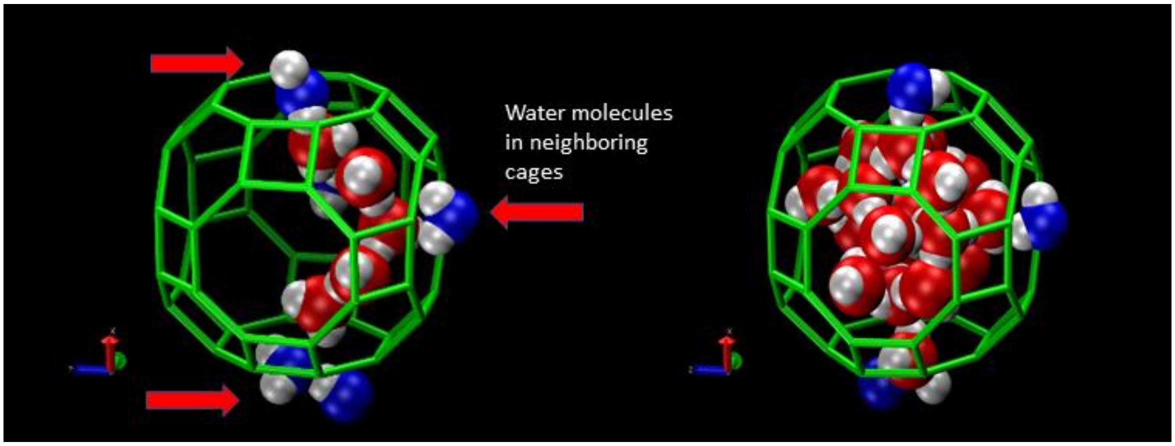

Figure S9. Molecular configurations representing early (left) and advanced (right) stages of water penetration into an initially empty cage. Red spheres indicate water molecules forming a hydrogen-bonded chain within the cage, stabilized by water molecules from neighboring filled cages (blue) via 8MR windows. At the advanced stage, water forms a globular cluster. Only neighboring molecules adjacent to the 8MR windows are shown.

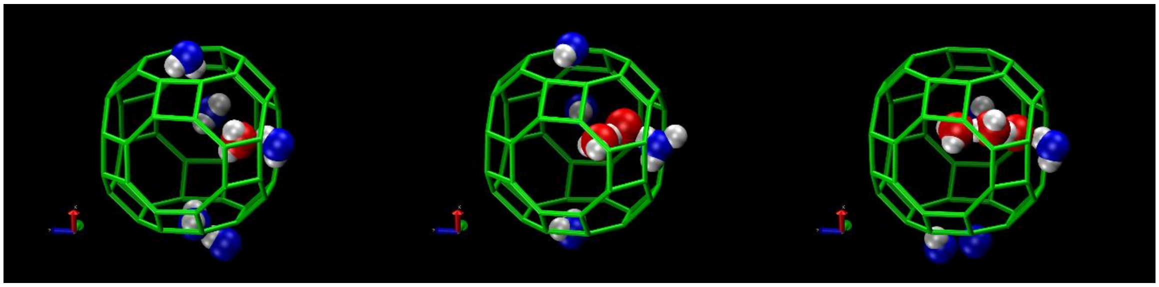

102 103 104

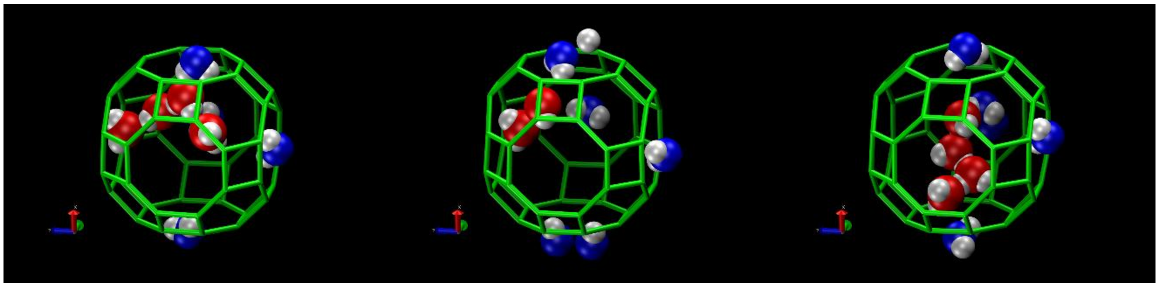

105 106 107

Figure S10. Temporal evolution of water penetration into an empty cage located in the core of the LTA grain, obtained according to Procedure 2 at 300 K and 80 MPa. Numbers indicate the configuration index (frame number), in the HISTORY file saved with a 40 ps timestep. Green lines connect silicon atoms forming the cage. Oxygen atoms of water molecules inside the cage are shown as red spheres, while those in adjacent cages are shown as blue spheres. The left and front neighboring cages remain empty during the productive run.

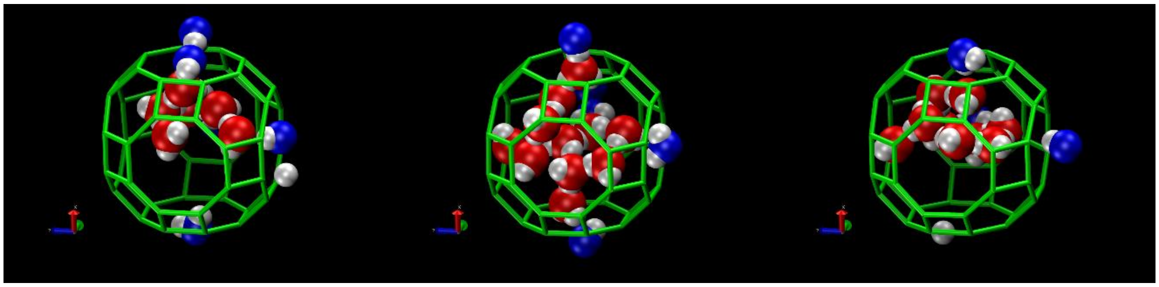

108 109 110

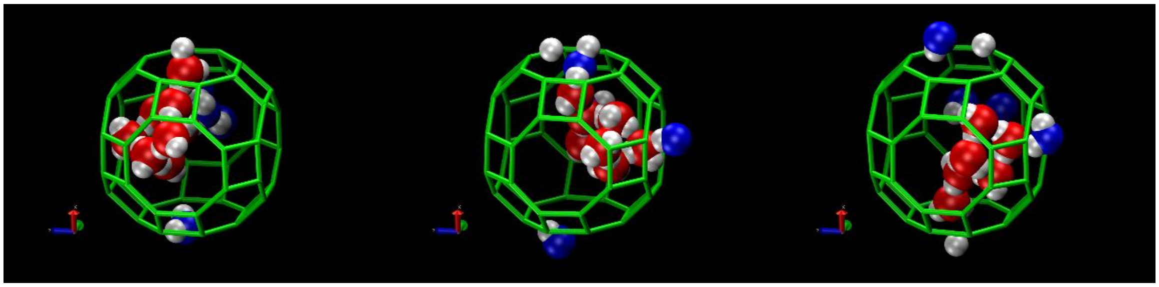

111 112 113

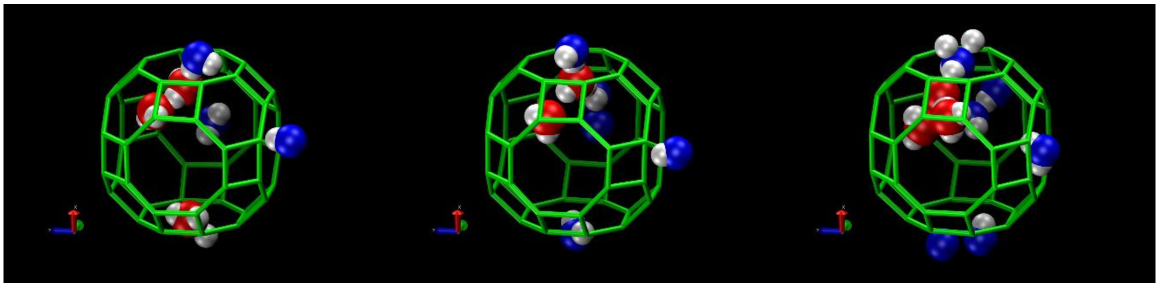

114 115 116

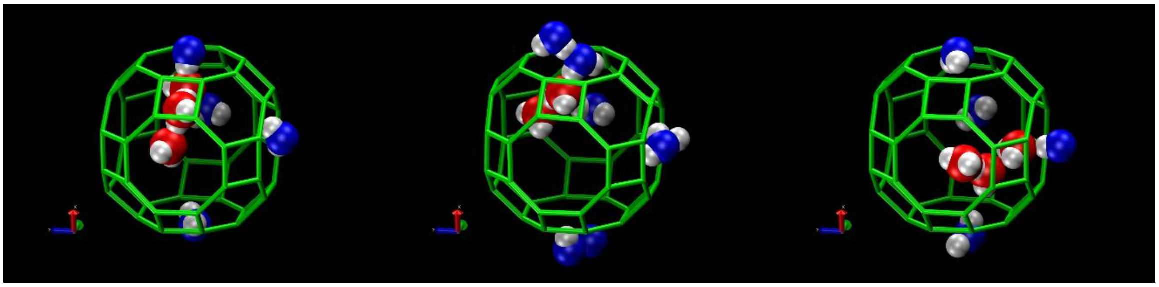

117 118 119

Figure S10. *Continued.*

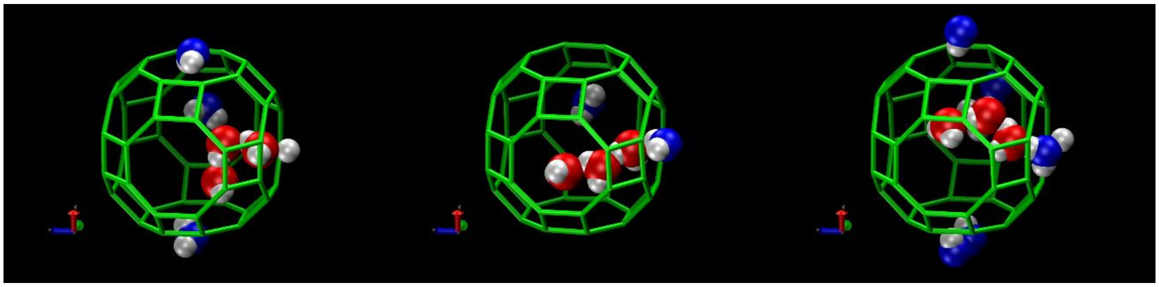

120 121 122

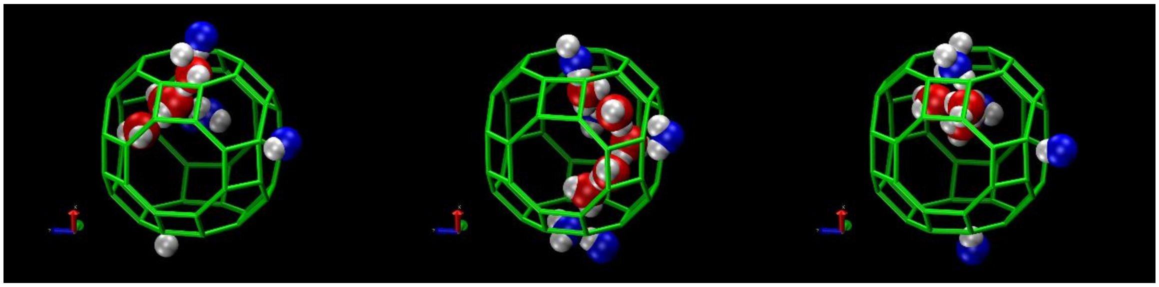

123 124 125

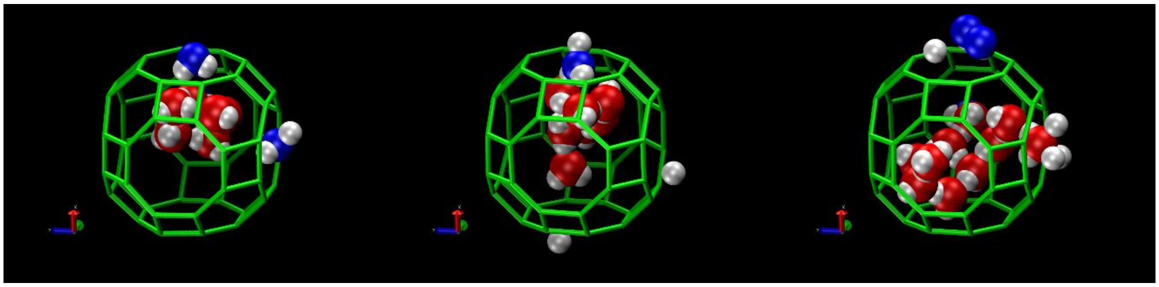

126 127 128

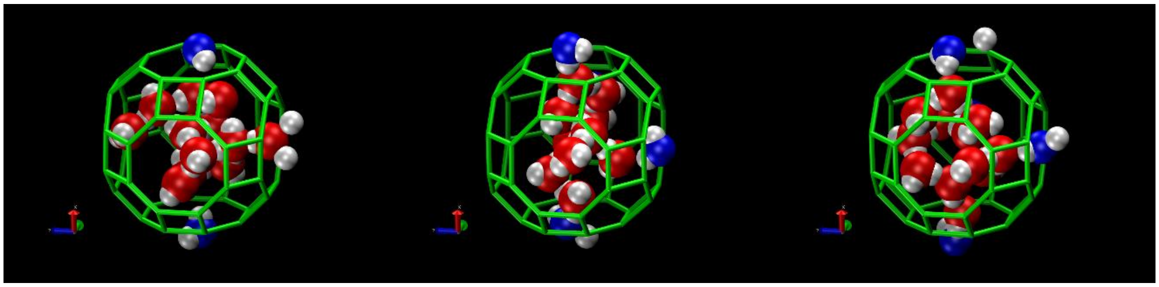

129 130 131

Figure S10. *Continued.*

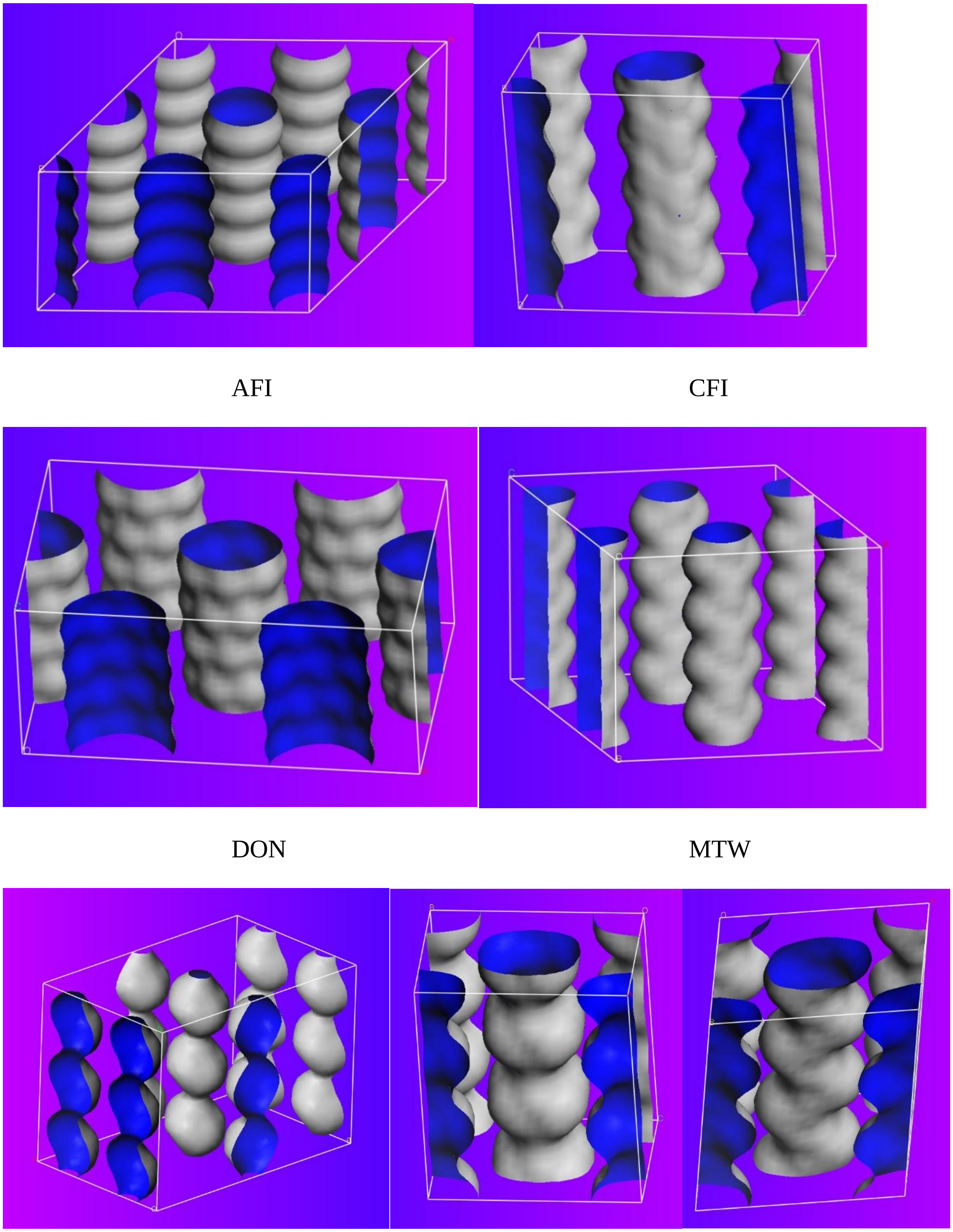

AFI CFI

DON MTW

MTF STF IFR

Figure S11. Schematic representation of pore architectures (not to scale) of zeolites with 1D channels (AFI, CFI, DON, MTW) and 1D channel systems containing side pockets (MTF, STF, IFR).

Table S2. Intrusion pressures and structural characteristics of zeolite pore systems. *A* denotes the Connolly accessible surface area, *V* the pore volume, and *d* the maximum diameter of a sphere able to diffuse through the pore system.

Topology	P_{int}, MPa	A, Å^2	V, Å^3	A/V, Å^{-1}	d, Å	$1/d$, Å^{-1}
1D channels						
TON	186	2945.0	3792.8	0.776	5.11	0.196
MTT	176	1446.8	1814.8	0.797	5.07	0.197
MTW	132	3379.5	4976.1	0.679	5.68	0.176
CFI	75	3139.4	4318.8	0.727	7.26	0.137
AFI	57	1784.5	3241.8	0.550	7.42	0.135
DON	26	523.6	1091.9	0.479	8.07	0.124
1D channels with side pockets						
MTF	125	2434.5	3015.3	0.807	4.03	0.248
STF	49	1556.7	2509.0	0.620	5.44	0.184
IFR	42	3178.7	5219.5	0.609	6.38	0.157
Cages						
DDR	60	13741.7	16583.6	0.829	3.65	0.274
STT	40	7121.3	10275.4	0.693	2.76	0.362
CHA	29	5287.0	8067.3	0.655	3.72	0.269
LTA	15	3678.8	6322.6	0.582	4.21	0.238

Description for Movies S1-S3

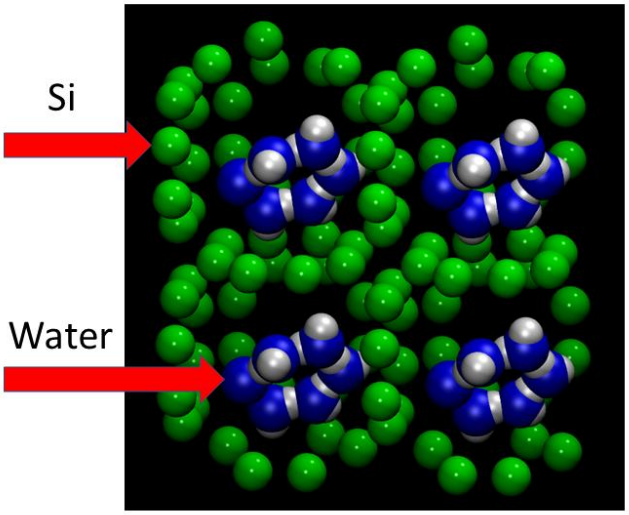

The movies depict the time evolution of water clusters within an LTA unit cell, simulated according to Procedure 1. The timestep between consecutive frames is 1 ps. Four identical cells are shown, and the front silicon atoms are omitted for clarity.
